# The effects of social media (Snapchat) interventions on the knowledge of oral health during pregnancy among pregnant women in Saudi Arabia

**DOI:** 10.1371/journal.pone.0281908

**Published:** 2023-02-16

**Authors:** Khalid Aboalshamat, Jomana Alharbi, Sharifah Alharthi, Alaa Alnifaee, Amal Alhusayni, Reem Alhazmi

**Affiliations:** 1 Dental Public Health Division, Preventative Dentistry Department, College of Dentistry, Umm Al-Qura University, Makkah, Saudi Arabia; 2 College of Dentistry, Umm Al-Qura University, Makkah, Saudi Arabia; Shahid Beheshti University of Medical Sciences, School of Dentistry, ISLAMIC REPUBLIC OF IRAN

## Abstract

**Background:**

There is growing interest in using social media to improve pregnant women’s well-being. This study aimed to evaluate the effects of social media (Snapchat) dissemination of health-promoting interventions on knowledge of oral health during pregnancy among pregnant women in Saudi Arabia.

**Materials and methods:**

Using a single-blinded parallel group randomized controlled trial design, 68 volunteers were assigned to either a study group (SG) or a control group (CG). The SG received information about oral health during pregnancy via Snapchat, while the CG received the same information using WhatsApp. The participants were assessed three times: T1 prior to the intervention, T2 immediately following the intervention, and T3 as a follow-up 1 month later.

**Results:**

A total of 63 participants completed the study in the SG or CG. According to paired t-test, total knowledge scores in the SG and CG increased significantly from T1 to T2 (*p* < 0.001) and from T1 to T3 (*p* < 0.001), but there was no significant change from T2 to T3 in either the SG or CG (*p*  =  0.699 and *p*  =  0.111, respectively). Using t-test, no significant differences were found between the SG and CG at T2 (*p*  =  0.263) or T3 (*p*  =  0.622). Also using t-test, no significant differences were found in the scores of the SG and CG from T2 to T1 (*p*  =  0.720), T3 to T2 (*p*  =  0.339), or T3 to T1 (*p*  =  0.969).

**Conclusions:**

Using social media (e.g., Snapchat and WhatsApp) as a health-promoting intervention is a promising method for improving women’s knowledge about oral health during pregnancy for short term. However, further studies are needed to compare social media with conventional standard lecturing methods. also, to assess the longevity of the impact (short or long term).

## Introduction

Social media is defined as any of a collection of applications that use the internet to create technical and ideological foundations that permit generating and sharing of content [1]. The public in general, and patients in particular, tend to seek health information from social media based on their interests, in addition to gaining emotional support from user interactions [2]. However, low reliability and the spreading of fake health information on social media have been cited in the literature as major concerns [2, 3], as reported in a recent systematic review [4]. Moreover, these issues became more serious and noticeable during the COVID-19 pandemic [5].

Pregnancy is a unique health phase in a woman’s life, as it is influenced by complicated physiological changes that can have a negative impact on the mother’s oral health such as pregnancy gingivitis, pregnancy granuloma, caries, xerostomia, and tooth mobility sometime [6]. Pregnant women have been found to seek health information during the perinatal period, throughout pregnancy, and after delivery [7]. However, in terms of dental health, many pregnant women tend to avoid seeing a dentist, although it can be a source of medical information and treatment [8], because they think they, or their fetus would be harmed as a result of the radiograph, dental procedures or medication. This illustrates the need to boost knowledge among pregnant women with information regarding oral health and dental treatment considerations during pregnancy.

There is growing interest in using social media and mobile health applications to improve pregnant women’s well-being [9]. A systematic review and meta-analysis showed several randomized controlled trial (RCT) and intervention studies aimed to improve different health aspects of pregnant women using social media and mobile applications [9, 10]. Another systematic review showed many interventional studies that have been used to improve oral health during pregnancy [11]. However, studies on the use of social media as an intervention are lacking. A search of the literature showed a single study that aimed to improve oral health during pregnancy using social media (video-sharing platform) [12]. This pre- and post-interventional study resulted in an improvement in pregnant women’s knowledge about dental visits, the prevention of caries, and bacteria transmission during pregnancy [12]. Nevertheless, this study did not have a comparison group to validate the improvement.

One of the most famous social media platforms is Snapchat. Snapchat is typically used to post selfie photos and videos among friends and family in a relaxed manner as a substitute for messaging [13]. In fact, Snapchat has been reported to be associated with bonding between users, rather than merely socializing [13]. One important feature of Snapchat is that videos are available for a short time before disappearing [14]. Snapchat is one of the most used social media platforms in Saudi Arabia, including females, and could be used as a medium to conduct health promotional activities. Despite the popularity of Snapchat, health-promoting activities using such platforms are scarce.

## Aim

The aim of this study was to evaluate the effects of social media (specifically Snapchat) health-promoting interventions on oral health knowledge during pregnancy among pregnant women.

## Materials and methods

### Study design and participants

**Fig 1:**
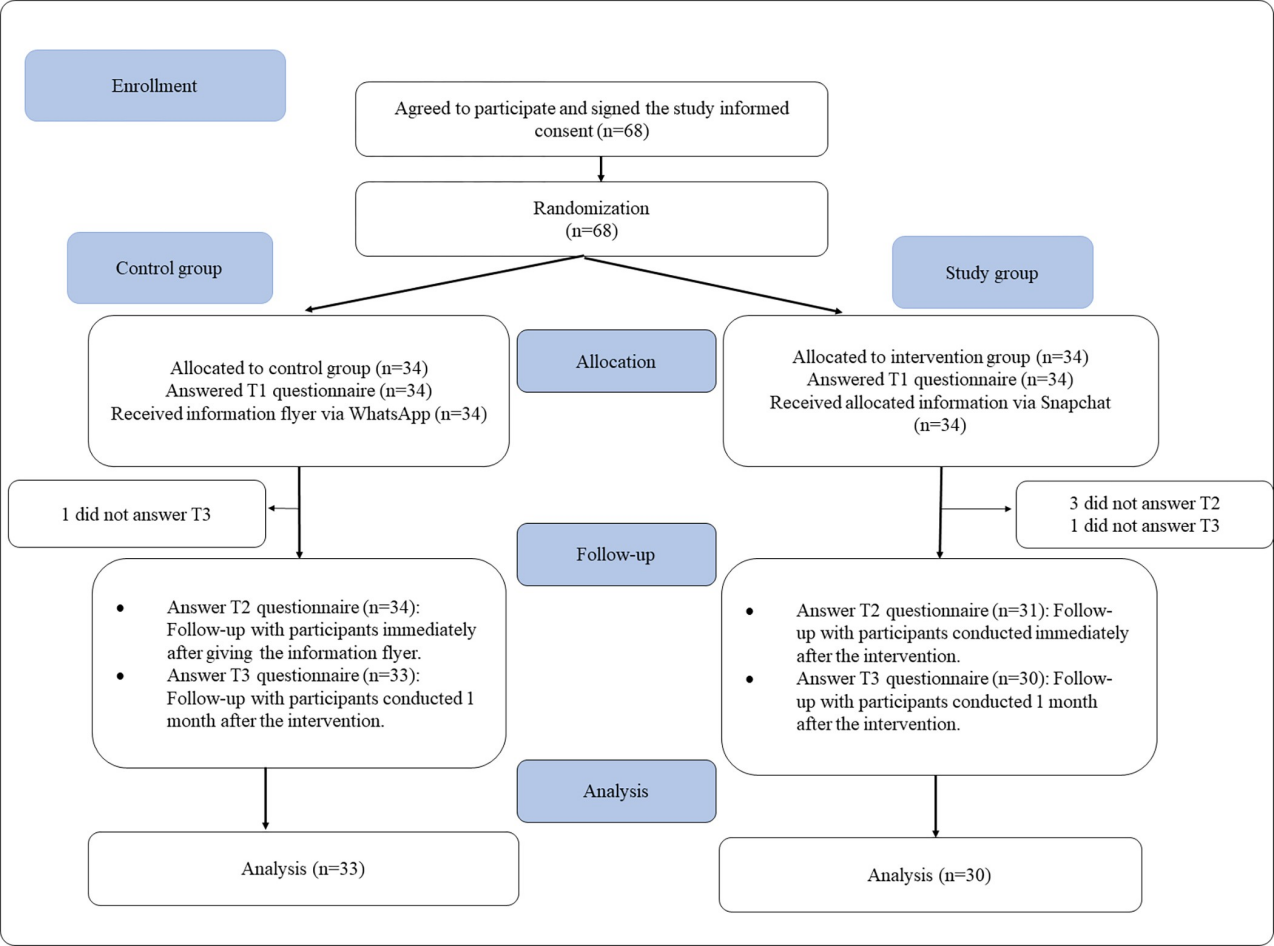
Participant interaction flowchart.

This study used a single-blinded parallel group RCT design in which study group (SG) participants received information about oral health during pregnancy via the Snapchat mobile application. Only participants were blinded, while examiners and statistician were not. The participants in the control group (CG) received the same information as a written flyer, as shown in "Fig 1".

CONSORT guidelines were used to document this study. The participants were all pregnant women living in Saudi Arabia (at any city), and they were recruited using convenience sampling through invitations to social media groups about pregnancy in Saudi Arabia. This includes WhatsApp, Twitter, and other social media platforms. The invitation included the study information and invited only pregnant women to respond to the invitation, by giving initial acceptance. Recruiting started at 20/11/2021. The inclusion criteria were (a) pregnant women in Saudi Arabia, who (b) spoke Arabic, (c) were able to use Snapchat and WhatsApp social media platforms, and (d) approve the study consent form. The participants did not know each other, and they were not treated before by the research team in the dental clinic, this to avoid the intervention contamination. The research team excluded all participants who did not sign the consent form that presented the study agreement, or anyone who was not fulfilling the other inclusion criteria The WhatsApp mobile application was used to communicate throughout the study because it is one of the most common messaging methods used in Saudi Arabia and is widely accepted. WhatsApp also has end-to-end encryption for maintaining participants’ confidentiality [15]. The sample size was calculated using RCT with two independent samples, continuous outcomes, and a two-tailed hypothesis formula [16]:

$$n(per\;group)=2{\left(\frac{Z_{1-\alpha /2}+Z_{\beta -1}}{ES}\right)}^{2}$$

$$ES={\left(\frac{minimal\;clinical\;difference}{standard\;devation}\right)}^{}$$

Where ES stands for effect size. As the study power of 90% was used, and a  =  0.05, B  =  0.1, constant Z(β−1)  =  1.282, and constant Z (1−a/2)  =  1.96. The standard deviation (SD) of a previous study on a related topic was 3.06 [17]. Three points were used as minimal clinical differences. According to the previous numbers, 21 participants were needed in each group, for a total of 42 participants in the study. The latter number was multiplied by 1.5 twice for the expected non-response rate and the design effect. This resulted in 90 invitations required for this study.

### Setting

After an initial agreement to participate in the study, an invitation was sent as a message to potential participants’ mobile phones using the WhatsApp platform, which allowed for communication between the participants and the research team and sending or receipt of the consent, intervention/CG flyer, and the online questionnaires.

The participants were randomly assigned to either the SG or the CG by the research team. The simple randomization process consisted of previously shuffled sealed envelopes with numbers with an equal allocation ratio placed in a bowl. Each participant randomly chose an envelope number virtually, giving them an equal chance of being placed in either group. The sealed envelopes were opaque and numbered in a sequence to ensure allocation concealment. To achieve blindness, the participants were told that the study’s goal was to examine two ways of information delivery meant to improve oral health knowledge among pregnant women. The research team used WhatsApp to give the participants a link that contained the study consent form and the baseline questionnaire (T1). Then, the participants in the SG received a barcode to add the intervention’s Snapchat account. The participants were assessed three times: T1 prior to the intervention, T2 immediately following the intervention (at the same day), and T3 for follow-up 1 month later to assess their retention of the information.

### Intervention and control

The trail started at 03/11/2021. Participants in the SG received information regarding oral health during pregnancy using the Snapchat mobile application. The oral health information was retrieved from previous studies [17–19], and the contents were validated by oral pathology and oral medicine consultants. The information included general information about dental treatments for pregnant women, recommended times to have dental treatments and emergencies, dental setting positions, recommendations for elective dental treatments, dental appointments, root canal treatment safety issues for pregnant women, radiographic precautions, local anesthesia, antibiotic usage, analgesics, pregnancy gingivitis, and methods of reducing periodontal problems. The intervention was delivered over the course of 2 weeks; each week had two Snapchat stories (a story is a series of short videos, each 10 seconds long, as permitted by the Snapchat application). Each story was around 5 to 10 minutes. Participants were able to submit questions regarding the material through the intervention Snapchat account.

The stories were presented as a diary of the research team members, like most of the stories on Snapchat. The research team performed roles within the clinic of the dental teaching hospital at Umm Al-Qura University for dental chair settings, precautions with radiographs, materials that are likely to be used during dental treatments, and demonstration of dental flossing and toothbrushing. The participants received the content spontaneously throughout the intervention days. At the same time, the CG received the same information in a written flyer sent only once via WhatsApp.

### Assessment

Questionnaires in a self-reported soft copy format were distributed to both groups at the three time points previously discussed: T1, T2, and T3. All identifiable data used to match participant data in T1, T2, and T3 were discarded after completing the T3 data collection. The questionnaires were identical for both groups at T1 and T3. At T2, the questionnaires were similar, but participants in the SG had an additional section assessing their experiences using Snapchat as a method of information delivery, while the CG received the same questions to assess the use of WhatsApp as a method of information delivery. The questionnaires contained sections that collected demographic information, use of social media, oral health knowledge among pregnant women, and experiences and perceptions of the social media intervention, as shown in "Table 1".

**Table 1:** Contents of questionnaire at T1, T2, and T3.

The time when the questionnaire was administered	Questionnaire content	
	Study group	Control group
T1 (before the intervention)	• Demographic information • Using social media • Oral health knowledge among pregnant women	• Demographic information • Using social media • Oral health knowledge among pregnant women
T2 (immediately after the intervention) at the same day	• Oral health knowledge among pregnant women • Experiences and perceptions of the social media (Snapchat) intervention	• Oral health knowledge among pregnant women • Experiences and perceptions of using WhatsApp as a comparison
T3 (1 month after the intervention)	• Oral health knowledge among pregnant women	• Oral health knowledge among pregnant women

The first part of the questionnaire included questions to collect demographic information on age, marital status, education level, family monthly income, regular visits to a dentist, number of children, month of current pregnancy, and sources of dental information. The second part included questions about the use of social media, average hours spent on social media per day, the most-used social media platform, and average hours spent on Snapchat and WhatsApp per day. The third part assessed knowledge about oral health and dental setting treatments for pregnant women with 20 questions answered with yes, no, or I do not know. Each question had only one correct answer, and the scores for correct answers were totaled into the total knowledge score, with the highest score of 20 points (highest level of knowledge) and the lowest score of zero (no knowledge at all). The research team formulated the questions based on the information provided in the intervention for the SG and CG. The last section assessed the participants’ experiences and perceptions of the social media used in the study (Snapchat or WhatsApp). This part contained 10 statements, with answers ranging from 1 (strongly disagree) to 5 (strongly agree). Most of the questions in this last section were adapted with modifications from a previous study [20]. All sections of the questionnaire were administered in Arabic.

The Snapchat videos and WhatsApp flyer were prepared two times before their final versions. The final version of the interventions, including the Snapchat videos, WhatsApp flyer, and questionnaire, went into a pilot study with 12 participants to validate the content, spelling, syntax, organization, clarity, grammar used in the questions, and audience comprehension. The content validation was done as the participants in the pilot were asked to the read the question, then to say it their way to be sure that the content is identical to the question content. The participants in the pilot round were not included in the main study analysis.

### Incentives and ethical considerations

After data collection at T3, all identifiable data were completely discarded. Participation was voluntary, and as an incentive, the participants were entered into six separate random prize drawings for 50 Saudi Riyals (USD 13.33) in the form of local bookstore gift cards. Before participating in the intervention, all participants have to approve the study’s informed consent form, by clicking “approve” on the electronic consent format. No witness was there to witness their approval, as their participation was done electronically. There are no minor participants in this study. The study was approved by the institutional review board of Umm Al-Qura University with the number HAPO-02-K-012-2021-11-810, which follows Declaration of Helsinki. This study was registered in the ISRCTN registry with the number ISRCTN13915540 (registration data 10/11/2021), which can be accessed at https://www.isrctn.com/ISRCTN13915540.

### Data analysis

The statistically significant level was set at 0.05. The data were gathered, tabulated, and statistically analyzed using SPSS software package version 27 (IBM Corp., Armonk, NY, USA). Chi-square, t-test, Fisher’s exact test, and paired t-test were used to analyze the data collected in this study. The family monthly income question had three categories based on previous researches conducted by the principal investigator on the Saudi population [21, 22].

## Results

A total of 90 participants were invited to this study, but only 68 agreed to participate (response rate  =  75.55%). The randomization process yielded 34 participants in the SG and a similar number in the CG. A total of 5 participants dropped out of the study, resulting in 30 participants in the SG and 33 participants in the CG, as shown in Fig 1, with a dropout rate of 7.35%. Only the data from the 63 participants were analyzed in this study. There were zero missing values because we used a mandatory electronic format questionnaire. The participants’ mean age was 28.67 years with SD of 4.71 years. All the participants (100%) were married women, with no divorced, widowed, or single participants. The demographic data for both groups are presented in "Table 2". The participants frequently used social media platforms are illustrated in "Table 3". According to the chi-square, Fisher’s exact test, and t-test, the demographic variables or variables about social media use in "Tables 2 and 3" were not statistically significant (*p* > 0.05).

**Table 2:** Demographic variables of the pregnant women in this study.

Demographic		Total	Study group (n = 30)	Control group (n = 33)
		*n* (%)	*n* (%)	*n* (%)
Age		m = 28.67 (SD = 4.71)	m = 28.33 (SD = 5.13)	m = 28.97 (SD = 4.36)
Number of children		m = 1.29 (SD = 1.37)	m = 1.17 (SD = 1.42)	m = 1.39 (SD = 1.34)
Education	High school	11 (17.5)	5 (16.7)	6 (18.2)
	Bachelor’s degree	46 (73.0)	21 (70)	25 (75.8)
	Higher education	6 (9.5)	4 (13.3)	2 (6.1)
Family income	<5,000 SAR	14 (22.2)	7 (23.3)	7 (21.2)
	5,000–15,000 SAR	44 (69.8)	22 (73.3)	22 (66.7)
	>15,000 SAR	5 (7.9)	1 (3.3)	4 (12.1)
Do you visit the dentist regularly?	Yes	7 (11.1)	4 (13.3)	3 (9.1)
	Only when needed	12 (19.0)	5 (16.7)	7 (21.2)
	No	44 (69.8)	21 (70)	23 (69.7)
Are you afraid of visiting the dentist?	Yes	24 (38.1)	13 (43.3)	11 (33.3)
	No	39 (61.9)	17 (56.7)	22 (66.7)
Pregnancy month		m = 5.51 (SD = 2.33)	m = 5.33 (SD = 2.50)	m = 5.67 (SD = 2.19)
First pregnancy?	Yes	24 (38.1)	14 (46.7)	10 (30.3)
	No	39 (61.9)	16 (53.3)	23 (69.7)
Source of oral or dental information:				
Doctors	Yes	32 (50.8)	16 (53.3)	16 (48.5)
	No	31 (49.2)	14 (46.7)	17 (51.5)
Dentists	Yes	55 (87.3)	26 (86.7)	29 (87.9)
	No	8 (12.7)	4 (13.3)	4 (12.1)
Social media	Yes	51 (81.0)	24 (80)	27 (81.8)
	No	12 (19.0)	6 (20)	6 (18.2)
Internet	Yes	56 (88.9)	28 (93.3)	28 (84.8)
	No	7 (11.1)	2 (6.7)	5 (15.2)
TV	Yes	12 (19.0)	4 (13.3)	8 (24.2)
	No	51 (81.0)	26 (86.7)	25 (75.8)
Books	Yes	11 (17.5)	4 (13.3)	7 (21.2)
	No	52 (82.5)	26 (86.7)	26 (78.8)
Other	Yes	18 (28.6)	11 (36.7)	7 (21.2)
	No	45 (71.4)	19 (63.3)	26 (78.8)

**Table 3:** Frequency of using social media by pregnant women in Saudi Arabia in the current study.

Question		Total	Study group (n = 30)	Control group (n = 33)
		*n* (%)	*n* (%)	*n* (%)
Do you use WhatsApp?	Yes	59 (93.7)	29 (96.7)	30 (90.9)
	No	4 (6.3)	1 (3.3)	3 (9.1)
Do you use Twitter?	Yes	35 (55.6)	20 (66.7)	15 (45.5)
	No	28 (44.4)	10 (33.3)	18 (54.5)
Do you use Snapchat?	Yes	60 (95.2)	29 (96.7)	31 (93.9)
	No	3 (4.8)	1 (3.3)	2 (6.1)
Do you use Instagram?	Yes	53 (84.1)	26 (86.7)	27 (81.8)
	No	10 (15.9)	4 (13.3)	6 (18.2)
Do you use TikTok?	Yes	31 (49.2)	15 (50)	16 (48.5)
	No	32 (50.8)	15 (50)	17 (51.5)
Do you use YouTube?	Yes	42 (66.7)	20 (66.7)	22 (66.7)
	No	21 (33.3)	10 (33.3)	11 (33.3)
Do you use Telegram?	Yes	29 (46.0)	16 (53.3)	13 (39.4)
	No	34 (54.0)	14 (46.7)	20 (60.6)
Average hours per day spent on social media		m = 4.84 (SD = 2.41)	m = 4.80 (SD = 1.94)	m = 4.88 (SD = 2.80)
Hours spent on Snapchat per day		m = 2.38 (SD = 1.54)	m = 2.50 (SD = 1.66)	m = 2.27 (SD = 1.44)
Hours spent on WhatsApp per day		m = 1.95 (SD = 1.79)	m = 1.87 (SD1.36)	m = 2.03 (SD = 2.13)
Do you often believe health information provided by social media influencers?	Yes	9 (14.3)	1 (3.3)	8 (24.2)
	Neutral	39 (61.9)	20 (66.7)	19 (57.6)
	No	15 (23.8)	9 (30)	6 (18.2)

"Table 4" shows the mean, SD, minimum total knowledge score, and maximum total knowledge score (which ranged from zero as the minimum and 20 as the maximum) at T1, T2, and T3. Also, the Cronbach’s alpha (reliability) of the knowledge questions was 0.717, 0.755 and 0.722 in T1, T2 and T3 respectively, which is considered as good reliability.

**Table 4:** Mean knowledge scores of the pregnant women in the study and control groups at T1, T2, and T3.

Group	Time period	Mean	SD	Minimum	Maximum
Study group (n = 30)	T1 Total	10.17	3.63	3	17
	T2 Total	16.03	3.49	7	20
	T3 Total	15.90	3.09	10	19
Control group (n = 30)	T1 Total	10.64	3.60	3	17
	T2 Total	16.91	2.53	6	20
	T3 Total	16.27	2.88	9	20

Using independent t-test, no significant differences were found between the SG and CG at T2 (t(61)  =  −1.148, *p*  =  0.263), and T3 (t(61)  =  −0.496, *p*  =  0.622).

The total knowledge score in the SG increased significantly from T1 to T2 with t(29)  =  −6.304, *p*  =  < 0.001, but there was no significant change from T1 to T2 with t(29)  =  0.39, *p*  =  0.699. The total knowledge score in the SG overall increased significantly from T1 to T3 with t(29)  =  −6.916, *p*  =  < 0.001. Similarly, in the CG, the total knowledge score increased significantly from T1 to T2 with t(32)  =  −9.417, *p*  =  < 0.001, but there was no significant change from T1 to T2 with t(32)  =  1.637, *p*  =  0.111. The total knowledge score in the SG overall increased significantly from T1 to T3 with t(32)  =  −8.984, *p*  =  < 0.001.

**Fig 2:**
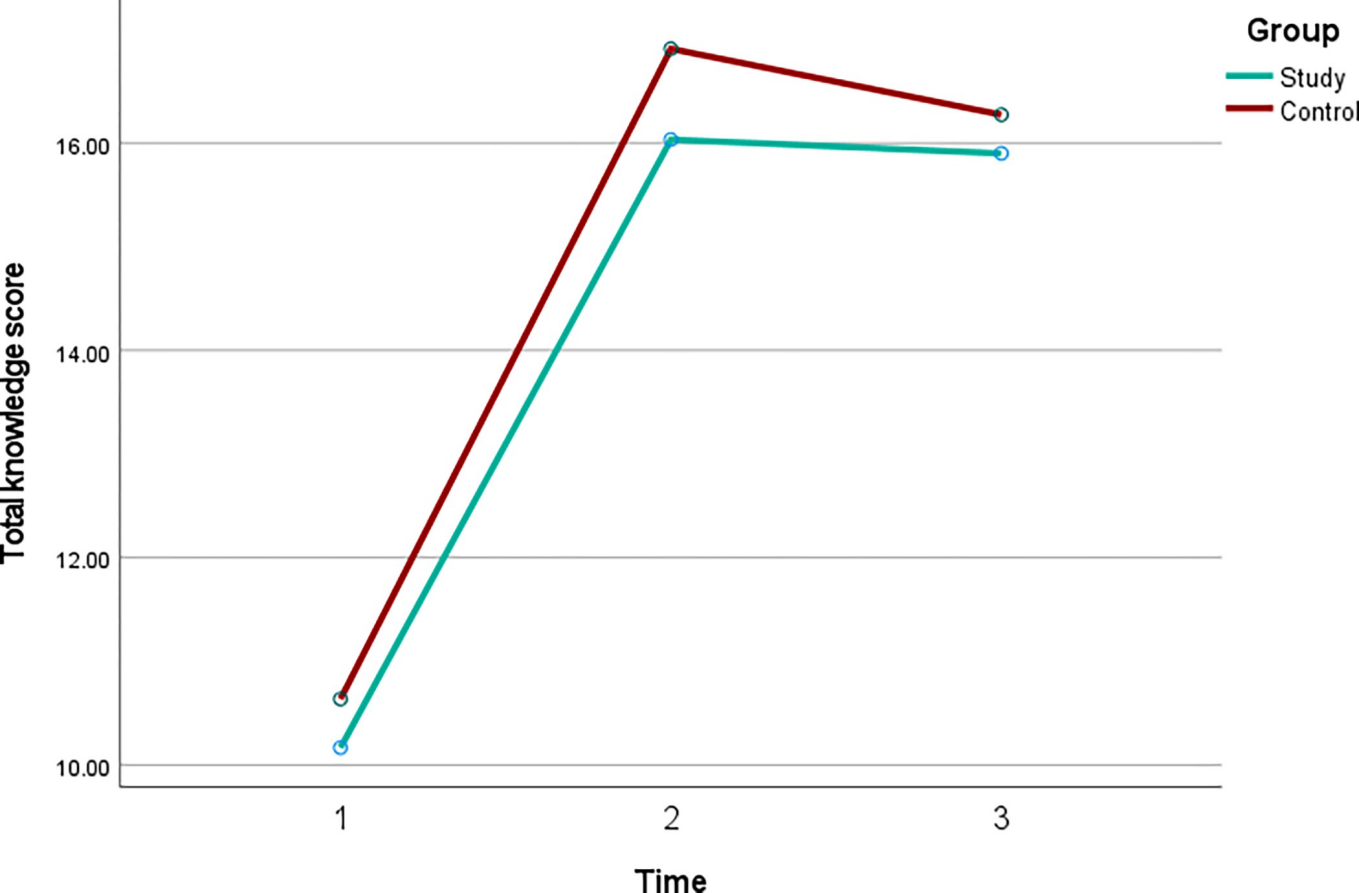
Changes in total knowledge scores over time.

"Table 5" shows that there were no significant differences in the scores between the SG and CG from T2 to T1, from T3 to T2, and from T3 to T1. This is also shown in "Fig 2".

**Table 5:** Differences in total knowledge scores of the pregnant women at T1, T2, and T3.

	Study group (n = 30)		Control group (n = 33)		*p*-value
	Mean	SD	Mean	SD	
T2_T1	5.87	5.1	6.27	3.83	0.720
T3_T2	−0.13	1.87	−0.64	2.23	0.339
T3_T1	5.73	4.54	5.64	3.6	0.969

"Table 6" shows the participants’ responses to the experience of receiving information by Snapchat for the SG or WhatsApp for the CG. According to a t-test, there were no significant differences between the scores in the groups for the two different applications.

**Table 6:** Study participants’ responses to receiving information in Snapchat (study group) or WhatsApp (control group).

Item	Study group (Snapchat) n = 30		Control group (WhatsApp) n = 33	
	Mean	SD	Mean	SD
I am generally fully satisfied with the health information I receive via this app.	3.3	1.73	3.61	1.68
Receiving oral health information via this app was helpful to me.	3.33	1.71	3.61	1.62
Receiving oral health information via this app was enjoyable.	3.27	1.62	3.39	1.66
Receiving oral health information via this app was innovative.	3.1	1.69	3.42	1.62
Accessing this app was easy.	3.27	1.72	3.73	1.64
The duration of this app’s promotional program was appropriate.	3.13	1.7	3.67	1.61
The information was easy to understand.	3.2	1.71	3.76	1.64
I advised pregnant women to receive oral health information through this app.	3.03	1.61	3.27	1.59
I will follow/join this app to transmit useful oral health information.	3.07	1.53	3.09	1.63
Knowing that the oral health information was reviewed scientifically was important to me.	3.2	1.65	3.76	1.58

## Discussion

The aim of this study was to evaluate social media (Snapchat) health-promoting interventions on knowledge about oral health during pregnancy among pregnant women in Saudi Arabia. The findings revealed that participants in both the study and control groups had significant improvements in their knowledge immediately after the intervention, and their retention of the information after 1 month was significantly better than their baseline knowledge. There was no significant difference between the SG and CG in knowledge improvement. The scores for the experience items regarding the intervention were higher than the midpoint for all items in both groups, but there was no significant difference between the SG and the CG on any item.

The results of previous interventional studies were similar to our study [12, 23, 24]. A previous study conducted in Dammam, Saudi Arabia [23], showed an improvement in health information using social media (Snapchat), similar to our study; however, there are four differences. First, our study used an active CG, while the prior study used a passive CG. Second, the intervention program for our study lasted for 2 weeks, while their intervention lasted for 4 weeks. Third, our study measured the retention of information after 1 month, while they did not follow up with their participants. Fourth, our study aimed to improve knowledge about a different topic (dental health during pregnancy versus breast cancer) among a different population (pregnant women). This might indicate that the social media platform Snapchat has the potential to raise awareness and improve knowledge about different health topics. However, our results cast doubts on the assumption that Snapchat is more effective than other social media platforms, such as WhatsApp, given that the results for both platforms were positive. Other studies in the United States and Iran [12, 24] indicated that social media is an effective method for increasing knowledge about oral health among pregnant women. However, the prior study used another social media platform (YouTube), which has wider accessibility than other social media platforms [25], and Telegram was also used as part of their intervention. This supports our finding that social media platforms are effective methods of boosting health-promoting interventions.

In the study design we planned to use WhatsApp to provide the same information among participants in the CG as an active comparison. This is because using WhatsApp in Saudi Arabia is observed to be very popular way to share information personally and even in work. Even health promotional materials are shared as text, flayer or infographic using WhatsApp, as form of digitalization of printed flyers used in the past years. In fact, printed flayers were one of the standard methods to increase the knowledge about health issue in Saudi Arabia. However, we did not consider using WhatsApp as another social media intervention. The main aim of our study was not to compare video contents to written contents, nor comparing the techniqual characteristics of Snapchat. However, the aim was to assess the experience of receiving the health information via Snapchat. This is because Snapchat have unique characteristics and unique interaction with audience [13] which is different than just watching a video. We could argue also that the experience of watching videos can induce different interactions within different social media platforms such as Snapchat, YouTube or TikTok. Also, it should be noticed that participants in the SG received the Snap chat stories several times, in compared to the control group participants who only received the message once. Also, it is not known if the duration on the intervention had positive or negative impact. This might induce a difference in the experience between the two groups. Such point can be taken into consideration in designing future intervention addressing the same topic.

It should be noted that researchers in Germany have found differences in how social media applications affect our health [26]. This might lead us to consider the influence of the usability of different social media applications that vary from one country to another [27]. This suggests horizons for future studies to assess different interventions using a wide array of social medical applications to investigate the differences in the same and different health-promoting interventions using various social media applications in different counties.

Because our results did not find a significant difference between the Snapchat and WhatsApp social media platforms, it can be supposed that there is not much difference in the effectiveness of one platform over another. However, it could be argued that the results of the CG are inflated. This is because the participants in the CG received the study’s flyer (one page) that contained all the intervention information that was stored on their devices. Thus, participants in the CG could refer back to the information to answer the questionnaire at T2 and T3 much easier than participants in the SG, where the information was found in the middle of Snapchat videos. In fact, a recent study found that WhatsApp was a good method of disseminating information in Pakistan about COVID-19 [28], which was counter to the findings of another study in Kuwait that limited the role of WhatsApp to social interaction needs with relatives and friends, in comparison to Snapchat, which focused on cognitive, affective, and personal interactive needs [29].

Based on the findings of this study and prior studies in the literature [12, 23, 24], we suggest that social media can be a useful tool for promoting education about oral health care. Moreover, social media has been found to be another method for teaching in health schools [30, 31], which opens new doors to using such powerful technology in positive ways. However, it should be noted that social media addiction and use have a negative aspect that needs to be taken into consideration during the designing of an intervention from an ethical point of view, as social media has been found to be related to psychological distress and poor sleep quality [32].

Additionally, we want to highlight that our intervention was simple and that the content used can be replaced by other content to enhance oral health education. Nevertheless, further research is recommended to validate this argument as it is not clear whether using social media as a venue to improve oral health is more effective than standard methods of disseminating information, such as lectures.

As the aim of this study was not to assess the prevalence of knowledge of pregnant women toward oral health knowledge, we did not include a thorough analysis about the questionnaire items and their results, which is available by the corresponding author upon request. The low sample size of the participants might give a false implication on emphasizing item by item discussion. Thus, we only included our discussion to the main aim of the study.

The strengths of this study include the study design, a single-blinded RCT, which is considered one of the most reliable study designs [33]. Only participants were blinded, and it was not possible to apply that to the examiners and statistician, because they were among the authors who designed the study and analyzed the study data. Also, this study is considered to be the first RCT in Saudi Arabia that aimed to evaluate the effects of social media (Snapchat) for improving oral health promotion among pregnant women. Unfortunately, there are a few limitations to be aware of, including a small sample size, short follow-up duration, and use of convenience sampling. All of these limitations reduce the external validity of the results for application to the general population. Furthermore, to validate the length of information retention, extra follow-up time is required. In fact, the current study does not answer the how long the effect of health promotion activity can last in compare to standard method. Also, the instrument content validity was not sufficient, as it should be repeated by domain experts regarding necessity, relevancy, simplicity, and clarity. Further studies are advisable with increases in the sample size and a random sample representing the general population for more generalizable results in Saudi Arabia. It is recommended that a study be conducted using different social media platforms and comparing them with a CG that receives standard conventional lectures and which has longer follow-up periods. It would also be desirable to use different types of information to be presented through social media to see how successful these platforms are with other aspects of oral health.

## Conclusion

Social media (Snapchat and WhatsApp) seem to be promising tools for promotional interventions to improve public health and dental knowledge among pregnant women, as well as with other health care topics. However, Snapchat is not superior to WhatsApp in terms of effectiveness. Snapchat is easy and popularly used among people with different socioeconomic statuses, and it was found to result in good retention of oral health knowledge for short term. Nevertheless, further studies are needed to generalize this study’s results, and to assess the longevity of such intervention (short- or long-term impact). This might give better insight to invest more in such interventions in the future.

## Supporting information

S1 File Study protocol in details. (PDF)

S2 File Study raw data. (PDF)

## List of abbreviations

CG: Control group
RCT: Randomized controlled trial
SD: Standard deviation
SG: Study group

## References

[1] Kaplan AM, Haenlein M. Users of the world, unite! The challenges and opportunities of social media. Bus Horiz. 2010;53(1):59–68.

[2] Zhao Y, Zhang J. Consumer health information seeking in social media: a literature review. Health Info Libr J. 2017;34(4):268–83. doi: 10.1111/hir.12192 29045011

[3] Zhou L, Zhang D, Yang CC, Wang Y. Harnessing social media for health information management. Electron Commer Res Appl. 2018;27:139–51. doi: 10.1016/j.elerap.2017.12.003 30147636 PMC6105292

[4] Melchior C, Oliveira M. Health-related fake news on social media platforms: a systematic literature review. New Media Soc. 2021;24(6):1500–22.

[5] Cinelli M, Quattrociocchi W, Galeazzi A, Valensise CM, Brugnoli E, Schmidt AL, et al. The COVID-19 social media infodemic. Sci Rep. 2020;10(1):16598. doi: 10.1038/s41598-020-73510-5 33024152 PMC7538912

[6] Steinberg BJ, Hilton IV, Iida H, Samelson R. Oral health and dental care during pregnancy. Dent Clin North Am. 2013;57(2):195–210. doi: 10.1016/j.cden.2013.01.002 23570802

[7] Baker B, Yang I. Social media as social support in pregnancy and the postpartum. Sex Reprod Healthc. 2018;17:31–4. doi: 10.1016/j.srhc.2018.05.003 30193717

[8] Jeihooni AK, Jamshidi H, Kashfi SM, Avand A, Khiyali Z. The effect of health education program based on health belief model on oral health behaviors in pregnant women of Fasa City, Fars Province, South of Iran. J Int Soc Prev Community Dent. 2017;7(6):336–43. doi: 10.4103/jispcd.JISPCD_339_17 29387617 PMC5774054

[9] Chan KL, Chen M. Effects of social media and mobile health apps on pregnancy care: meta-analysis. JMIR Mhealth Uhealth. 2019;7(1):e11836. doi: 10.2196/11836 30698533 PMC6372934

[10] Overdijkink SB, Velu AV, Rosman AN, van Beukering MD, Kok M, Steegers-Theunissen RP. The usability and effectiveness of mobile health technology-based lifestyle and medical intervention apps supporting health care during pregnancy: systematic review. JMIR Mhealth Uhealth. 2018;6(4):e109. doi: 10.2196/mhealth.8834 29691216 PMC5941088

[11] Vamos CA, Thompson EL, Avendano M, Daley EM, Quinonez RB, Boggess K. Oral health promotion interventions during pregnancy: a systematic review. Community Dent Oral Epidemiol. 2015;43(5):385–96. doi: 10.1111/cdoe.12167 25959402

[12] Bates SB, Riedy CA. Changing knowledge and beliefs through an oral health pregnancy message. J Public Health Dent. 2012;72(2):104–11. doi: 10.1111/j.1752-7325.2011.00289.x 22316424

[13] Piwek L, Joinson A. “What do they Snapchat about?” Patterns of use in time-limited instant messaging service. Comput Human Behav. 2016;54:358–67.

[14] Cortis Mack C, Harding M, Davies N, Ward G. RECAPP-XPR: a smartphone application for presenting and recalling experimentally controlled stimuli over longer timescales. Behav Res Methods. 2019;51(4):1804–23. doi: 10.3758/s13428-018-1157-x 30536149 PMC6690863

[15] Endeley RE. End-to-end encryption in messaging services and national security—case of WhatsApp messenger. J Inf Secur. 2018;9(1):95–9.

[16] Sullivan LM. Essentials of biostatistics in public health. 2nd ed. Sudbury: Jones & Bartlett Learning; 2011.

[17] Aboalshamat K, Abdulrahman S, Alowadi J, Al-Mutairy N, Fairak M, Alraithi N, et al. Endodontic treatment in pregnancy: knowledge, attitudes, and practices of dentists and interns in Jeddah, Saudi Arabia. Open Dent J. 2020;14(1):211–8.

[18] American Dental Association. Pregnancy. 2021. https://www.ada.org/resources/research/science-and-research-institute/oral-health-topics/pregnancy. Accessed 14 Aug 2022.

[19] American Pregnancy Association. Pregnancy and dental work. 2021. https://americanpregnancy.org/healthy-pregnancy/is-it-safe/dental-work-and-pregnancy/. Accessed 14 Aug 2022.

[20] Aboalshamat K, Khayat A, Halwani R, Bitan A, Alansari R. The effects of gamification on antimicrobial resistance knowledge and its relationship to dentistry in Saudi Arabia: a randomized controlled trial. BMC Public Health. 2020;20(1):680. doi: 10.1186/s12889-020-08806-2 32404076 PMC7222482

[21] Borajy S, Albkhari D, Turkistani H, Altuwairiqi R, Aboalshamat K, Altaib T, Almehman W. Relationship of electronic device usage with obesity and speech delay in children. Fam Med Prim Care Rev. 2019;21:93–7.

[22] Aboalshamat K, Aljrary D, Damanhuri R, Alnafisah A, Alghuraybi S, Aljifry S, et al. Preferred Obstetrician-gynaecologist Gender among Female Residents in Jeddah, Saudi Arabia. J. Umm Al-Qura Univ. Med Sci. 2020;6:21–5.

[23] Alanzi TM, Alobrah A, Alhumaidi R, Aloraifi S. Evaluation of the Snapchat mobile social networking application for breast cancer awareness among Saudi students in the Dammam region of the Kingdom of Saudi Arabia. Breast Cancer (Dove Med Press). 2018;10:113–9. doi: 10.2147/BCTT.S166135 30034251 PMC6047612

[24] Deghatipour M, Ghorbani Z, Mokhlesi AH, Ghanbari S, Namdari M. Effect of oral health promotion interventions on pregnant women dental caries: a field trial. BMC Oral Health. 2022;22:280. doi: 10.1186/s12903-022-02292-1 35804346 PMC9270746

[25] Brar J, Khalid A, Ferdous M, Abedin T, Turin TC. Breast cancer screening literacy information on online platforms: a content analysis of YouTube videos. Breast Dis. 2022;41(1):81–7. doi: 10.3233/BD-201028 34487015

[26] Rozgonjuk D, Sindermann C, Elhai JD, Montag C. Comparing smartphone, WhatsApp, Facebook, Instagram, and Snapchat: which platform elicits the greatest use disorder symptoms? Cyberpsychol Behav Soc Netw. 2021;24(2):129–34. doi: 10.1089/cyber.2020.0156 32907403

[27] Aboalshamat K, Alkiyadi S, Alsaleh S, Reda R, Alkhaldi S, Badeeb A, et al. Attitudes toward social media among practicing dentists and dental students in clinical years in Saudi Arabia. Open Dent J. 2019;13(1):143–9.

[28] Islam T, Mahmood K, Sadiq M, Usman B, Yousaf SU. Understanding knowledgeable workers’ behavior toward COVID-19 information sharing through WhatsApp in Pakistan. Front Psychol. 2020;11:572526. doi: 10.3389/fpsyg.2020.572526 33117239 PMC7575735

[29] Sultan AJ. Usage behaviors on Snapchat, Instagram, and WhatsApp: between-group analyses. International Journal of E-Services and Mobile Applications (IJESMA). 2021;13(1):45–59.

[30] Rajeh MT, Sembawa SN, Nassar AA, Al Hebshi SA, Aboalshamat KT, Badri MK. Social media as a learning tool: dental students’ perspectives. J Dent Educ. 2021;85(4):513–20. doi: 10.1002/jdd.12478 33219515

[31] Rajeh MT, Aboalshamat KT, Nassar AA, Sembawa SN, Al Hebshi SA, Badri MK. Insights on using social media in dental education: a cross-sectional study in Saudi Arabia. Open Dent J. 2020;14(1):717–23.

[32] Rajeh MT, Mahmoud MA, Badawoud AM, Alzhrani AM, Abdouh IM, Badri HM, Quronfulah BS. The effect of social media addiction on psychological distress, sleep quality and loneliness among health care professional in Saudi Arabia. Med Sci. 2022;26(125):1–9.

[33] Zabor EC, Kaizer AM, Hobbs BP. Randomized controlled trials. Chest. 2020;158(1):S79–S87. doi: 10.1016/j.chest.2020.03.013 32658656 PMC8176647

