# Supplementary material for: The effects of social media (Snapchat) interventions on the knowledge of oral health during pregnancy among pregnant women in Saudi Arabia
Source: PLoS One. 2023 Feb 16;18(2):e0281908. doi: 10.1371/journal.pone.0281908 (PMC9934359; doi:10.1371/journal.pone.0281908)
Supplement: S1 File — (PDF) [file pone.0281908.s001.pdf]

The effects of social media (Snapchat) interventions on the knowledge of oral health during pregnancy among pregnant women in Saudi Arabia: A randomized controlled trial

Study protocol

Project summary

This study aimed to evaluate the effects of social media (Snapchat) dissemination of health-promoting interventions on knowledge of oral health during pregnancy among pregnant women in Saudi Arabia.

General information

Protocol title: The effects of social media (Snapchat) interventions on the knowledge of oral health during pregnancy among pregnant women in Saudi Arabia: A randomized controlled trial

Name and address of the sponsor/funder: Self-funded.

Name and title of the investigator(s):

Khalid Aboalshamat[1], Jomana Alharbi[2], Sharifah Alharthi[2], Alaa Alnifaee[2], Amal Alhusayni[2], Reem Alhazmi[2]

[1]Dental Public Health Division, Preventative Dentistry Department, College of

Dentistry, Umm Al-Qura University, Makkah, Saudi Arabia.

[2]College of Dentistry, Umm Al-Qura University, Makkah, Saudi Arabia.

KA, SA, JA, AlA, AmA, and RA participated in the research idea, designing of the study, design the intervention, explaining of the results, and drafting of the

manuscript. SA, JA, AlA and AmA, conducted the intervention and obtained the data. KA did the statistical data analysis. SA, JA, AlA, AmA, and RA participated in data analysis steps. The final manuscript was read and approved by all KA, SA, JA, AlA, AmA, and RA.

Name(s) and address(es) of the clinical laboratory(ies) and other medical and/or technical department(s) and/or institutions involved in the research: Umm Al-Qura University, Taif Road, 21955, Mecca, Makkah Province, Saudi Arabia, +966 12 527 0000

Rationale & background information

Social media is defined as any of a collection of applications that use the internet to create technical and ideological foundations that permit generating and sharing of content [1]. The public in general, and patients in particular, tend to seek health information from social media based on their interests, in addition to gaining emotional support from user interactions [2]. However, low reliability and the spreading of fake health information on social media have been cited in the literature as major concerns [2, 3], as reported in a recent systematic review [4]. Moreover, these issues became more serious and noticeable during the COVID-19 pandemic [5].

Pregnancy is a unique health phase in a woman's life, as it is influenced by complicated physiological changes that can have a negative impact on the mother's oral health [6]. Pregnant women have been found to seek health information during the perinatal period, throughout pregnancy, and after delivery [7]. However, in terms of dental health, many pregnant women tend to avoid seeing a dentist, although it can be a source of medical information and treatment, because they believe they would be harmed [8]. This illustrates the need to boost knowledge among pregnant women with information regarding oral health and dental treatment considerations during pregnancy.

There is growing interest in using social media and mobile health applications to improve pregnant women's well-being [9]. A systematic review and meta-analysis showed several randomized controlled trial (RCT) and intervention studies aimed to improve different health aspects of pregnant women using social media and mobile applications [9, 10]. Another systematic review showed many interventional studies that have been used to improve oral health during pregnancy [11]. However, studies on the use of social media as an intervention are lacking. A search of the literature showed a single study that

aimed to improve oral health during pregnancy using social media (video-sharing platform) [12]. This pre- and post-interventional study resulted in an improvement in pregnant women's knowledge about dental visits, the prevention of caries, and bacteria transmission during pregnancy [12]. Nevertheless, this study did not have a comparison group to validate the improvement.

One of the most famous social media platforms is Snapchat. Snapchat is typically used to post selfie photos and videos among friends and family in a relaxed manner as a substitute for messaging [13]. In fact, Snapchat has been reported to be associated with bonding between users, rather than merely socializing [13]. One important feature of Snapchat is that videos are available for a short time before disappearing [14]. Despite the popularity of Snapchat, health-promoting activities using such platforms are scarce.

Methods

Aim

This study aimed to evaluate the effects of social media (Snapchat) dissemination of health-promoting interventions on knowledge of oral health during pregnancy among pregnant women in Saudi Arabia.

Study design and participants

This study used a single-blinded parallel group RCT design in which study group (SG) participants received information about oral health during pregnancy via the Snapchat mobile application. The participants in the control group (CG) received the same information as a written flyer, as shown in Fig. 1.

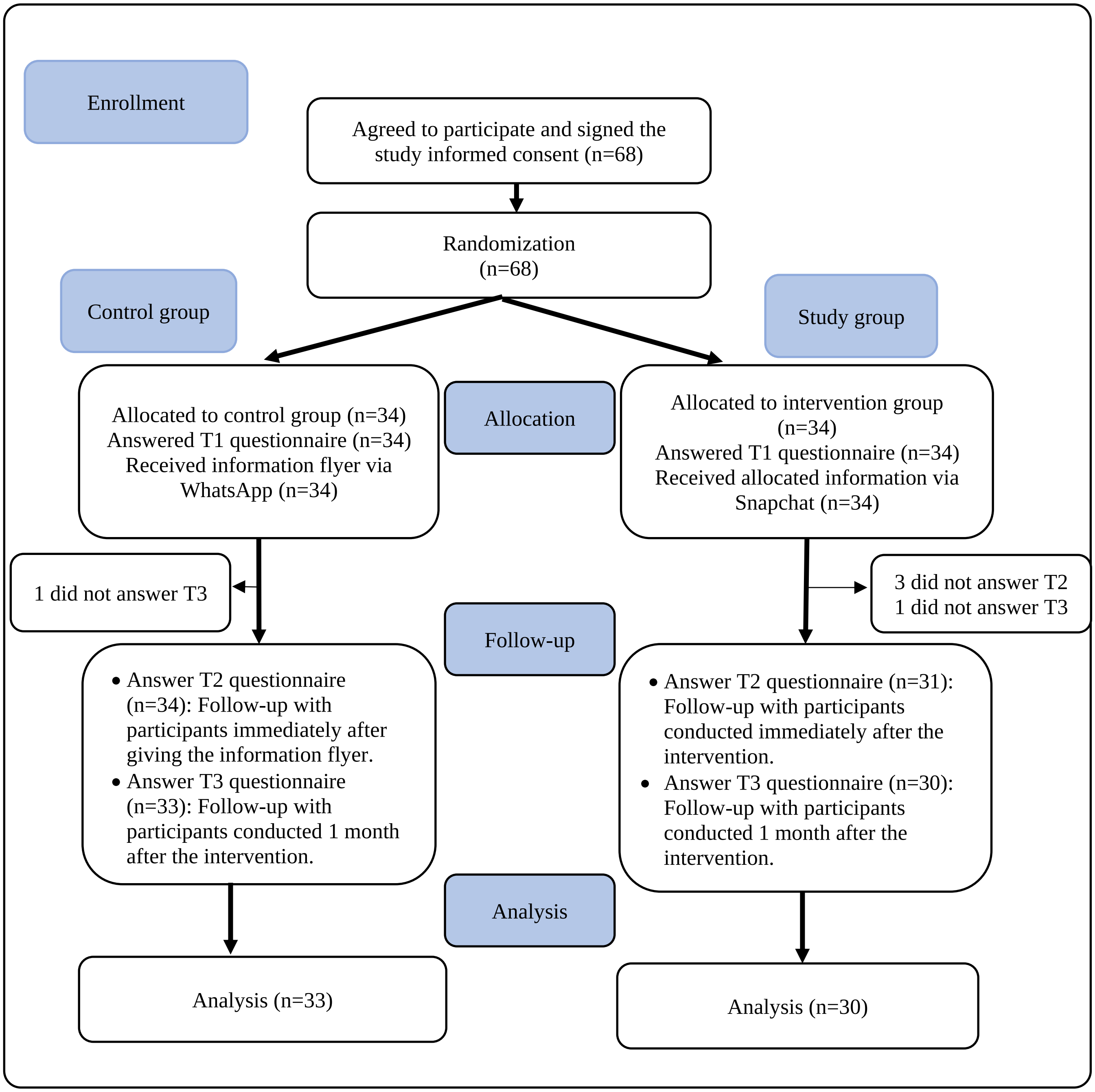

Fig. 1 Participant interaction flowchart

CONSORT guidelines were used to document this study. The participants were all pregnant women living in Saudi Arabia, and they were recruited using convenience sampling from Umm Al-Qura University dental hospital and through invitations to social media groups about pregnancy in Saudi Arabia. Recruiting started at 20/11/2021. The inclusion criteria were (a) pregnant women in Saudi

Arabia, who (b) spoke Arabic and (c) were able to use Snapchat and WhatsApp social media platforms. The research team excluded all participants who did not sign the consent form that presented the study agreement. The WhatsApp mobile application was used to communicate throughout the study because it is one of the most common messaging methods used in Saudi Arabia and is widely accepted. WhatsApp also has end-to-end encryption for maintaining participants' confidentiality [15]. The sample size was calculated using RCT with two independent samples, continuous outcomes, and a two-tailed hypothesis formula [16]:

$$n(per\ group) = 2\left(\frac{Z_{1-\alpha/2} + Z_{\beta-1}}{ES}\right)^2$$

$$ES = \left(\frac{\text{minimal clinical difference}}{standard\ devation}\right)$$

where ES stands for effect size. As the study power of 90% was used, and a = 0.05, B = 0.1, constant Z(β−1) = 1.282, and constant Z (1−a/2) = 1.96. The standard deviation (SD) of a previous study on a related topic was 3.06 [17]. Three points were used as minimal clinical differences. According to the previous numbers, 21 participants were needed in each group, for a total of 42 participants in the study. The latter number was multiplied by 1.5 twice for the expected non-response rate and the design effect. This resulted in 90 invitations required for this study.

Setting

After an initial agreement to participate in the study, an invitation was sent as a message to potential participants' mobile phones using the WhatsApp platform, which allowed for communication between the participants and the research team and sending or receipt of the consent, intervention/CG flyer, and the online questionnaires.

The participants were randomly assigned to either the SG or the CG by the research team. The simple randomization process consisted of previously shuffled sealed envelopes numbers with an equal allocation ratio placed in a bowl. Each participant randomly chose an envelope number virtually, giving them an equal chance of being placed in either group. The sealed envelopes were

opaque and numbered in a sequence to ensure allocation concealment. To achieve blindness, the participants were told that the study's goal was to examine two ways of information delivery meant to improve oral health knowledge among pregnant women. The research team used WhatsApp to give the participants a link that contained the study consent form and the baseline questionnaire (T1). Then, the participants in the SG received a barcode to add the intervention's Snapchat account. The participants were assessed three times: T1 prior to the intervention, T2 immediately following the intervention, and T3 for follow-up 1 month later to assess their retention of the information.

Intervention and control

Participants in the SG received information regarding oral health during pregnancy using the Snapchat mobile application. The oral health information was retrieved from previous studies [17–19], and the contents were validated by oral pathology and oral medicine consultants. The information included general information about dental treatments for pregnant women, recommended times to have dental treatments and emergencies, dental setting positions, recommendations for elective dental treatments, dental appointments, root canal treatment safety issues for pregnant women, radiographic precautions, local anesthesia, antibiotic usage, analgesics, pregnancy gingivitis, and methods of reducing periodontal problems. The intervention was delivered over the course of 2 weeks; each week had two Snapchat stories (a story is a series of short videos, each 10 seconds long, as permitted by the Snapchat application). Each story was around 5 to 10 minutes. Participants were able to submit questions regarding the material through the intervention Snapchat account.

The stories were presented as a diary of the research team members, like most of the stories on Snapchat. The research team performed roles within the clinic of the dental teaching hospital at Umm Al-Qura University for dental chair settings, precautions with radiographs, materials that are likely to be used during dental treatments, and demonstration of dental flossing and toothbrushing. The participants received the content spontaneously throughout the intervention days.

At the same time, the CG received the same information in a written flyer sent only once via WhatsApp.

Assessment

Questionnaires in a self-reported soft copy format were distributed to both groups at the three time points previously discussed: T1, T2, and T3. All identifiable data used to match participant data in T1, T2, and T3 were discarded after completing the T3 data collection. The questionnaires were identical for both groups at T1 and T3. At T2, the questionnaires were similar, but participants in the SG had an additional section assessing their experiences using Snapchat as a method of information delivery, while the CG received the same questions to assess the use of WhatsApp as a method of information delivery. The questionnaires contained sections that collected demographic information, use of social media, oral health knowledge among pregnant women, and experiences and perceptions of the social media intervention, as shown in Table 1.

Table 1 Contents of questionnaire at T1, T2, and T3

Questionnaire time	Study group	Control group
T1 (before the intervention)	• Demographic information<br>• Using social media<br>• Oral health knowledge among pregnant women	• Demographic information<br>• Using social media<br>• Oral health knowledge among pregnant women
T2 (immediately after the intervention)	• Oral health knowledge among pregnant women<br>• Experiences and perceptions of the social media (Snapchat) intervention	• Oral health knowledge among pregnant women<br>• Experiences and perceptions of using WhatsApp as a comparison
T3 (1 month after the intervention)	• Oral health knowledge among pregnant women	• Oral health knowledge among pregnant women

The first part of the questionnaire included questions to collect demographic information on age, marital status, education level, family monthly income, regular visits to a dentist, number of children, month of current pregnancy, and sources of dental information. The second part included questions about the use of social media, average hours spent on social media

per day, the most-used social media platform, and average hours spent on Snapchat and WhatsApp per day. The third part assessed knowledge about oral health and dental setting treatments for pregnant women with 20 questions answered with yes, no, or I do not know. Each question had only one correct answer, and the scores for correct answers were totaled into the total knowledge score, with the highest score of 20 points (highest level of knowledge) and the lowest score of zero (no knowledge at all). The research team formulated the questions based on the information provided in the intervention for the SG and CG. The last section assessed the participants' experiences and perceptions of the social media used in the study (Snapchat or WhatsApp). This part contained 10 statements, with answers ranging from 1 (strongly disagree) to 5 (strongly agree). Most of the questions in this last section were adapted with modifications from a previous study [20]. All sections of the questionnaire were administered in Arabic.

The Snapchat videos and WhatsApp flyer were prepared two times before their final versions. The final version of the interventions, including the Snapchat videos, WhatsApp flyer, and questionnaire, went into a pilot study with 12 participants to validate the content, spelling, syntax, organization, clarity, grammar used in the questions, and audience comprehension. The participants in the pilot round were not included in the main study analysis.

Incentives and ethical considerations

After data collection at T3, all identifiable data were completely discarded. Participation was voluntary, and as an incentive, the participants were entered into six separate random prize drawings for 50 Saudi Riyals (USD 13.33) in the form of local bookstore gift cards. Before participating in the intervention, all participants have to approve the study's informed consent form, by clicking "approve" on the electronic consent format. No witness was there to witness their approval, as their participation was done electronically. The study was approved by the institutional review board of Umm Al-Qura University with the number HAPO-02-K-012-2021-11-810, which follows Declaration of Helsinki. Additionally, the RCT was registered in the ISRCTN registry with the number ISRCTN13915540 (registration data 10/11/2021), which can be accessed at https://www.isrctn.com/ISRCTN13915540.

Data analysis and data management

The statistically significant level was set at 0.05. The data were gathered, tabulated, and statistically analyzed using SPSS software package version 27 (IBM Corp., Armonk, NY, USA). Chi-square, t-test, Fisher's exact test, and paired t-test were used to analyze the data collected in this study. The data were kept in a private computer, that is accessed by the PI at Umm Al-Qura University, which is protected by password. There was no missing data, because all online questions were compulsory.

Safety considerations

Any participant can withdraw from the study without any condition if she feel uncomfortable or distressed by any mean.

Follow-up

Participants were aware of the follow up after a month of the intervention as found in the consent form.

Dissemination of results and publication policy

Data will be published as an article in a respected journal in the field of oral health.

Project management

KA, SA, JA, AlA, AmA, and RA participated in the research idea, designing of the study, design the intervention, explaining of the results, and drafting of the manuscript. SA, JA, AlA and AmA, conducted the intervention and obtained the data. KA did the statistical data analysis. SA, JA, AlA, AmA, and RA participated in data analysis steps. The final manuscript was read and approved by all KA, SA, JA, AlA, AmA, and RA.

Budget

Item	Cost in Saudi Riyal (SAR): 1 SAR= 0.27 United States Dollar)
Gift vouchers	300 SAR
English editing	670 SAR
Publication fees	9000 SAR

Problems anticipated

There were some delay in response of some participants. This was handled by reminders to them. Also, few participants dropped out of the study.

Research team credentials

Khalid Aboalshamat[1], Jomana Alharbi[2], Sharifah Alharthi[2], Alaa Alnifaee[2], Amal Alhusayni[2], Reem Alhazmi[2]

[1]Dental Public Health Division, Preventative Dentistry Department, College of

Dentistry, Umm Al-Qura University, Makkah, Saudi Arabia.

[2]College of Dentistry, Umm Al-Qura University, Makkah, Saudi Arabia.

Other research activities of the principle investigators

The effect of smartphone addiction on psychological distress, sleep quality and loneliness among health care professional in Saudi Arabia.

The Experience of Teledentistry Among Follow-Up Patients in the Oral and Maxillofacial Surgery Clinics.
Artificial intelligence (AI) perceptional and utilization among dental professional in Saudi Arabia
The influence of smartphone addiction, social media addiction and sleep quality on satisfaction with life among health care professional in Saudi Arabia.
The effect of social media addiction on burnout and satisfaction with life among health care professional in Saudi Arabia.
The effect of sleep quality and psychological distress and satisfaction with life among health care professional in Saudi Arabia.
The relation of social media addiction, psychological distress and loneliness with suicidal ideation among health care professional in Saudi Arabia.
The effect of social media addiction on psychological distress, sleep quality and loneliness among health care professional in Saudi Arabia.

No funding agencies were involved

Reference

1. Kaplan AM, Haenlein M. Users of the world, unite! The challenges and opportunities of social media. Bus Horiz. 2010;53(1):59–68.

2. Zhao Y, Zhang J. Consumer health information seeking in social media: a literature review. Health Info Libr J. 2017;34(4):268–83.

3. Zhou L, Zhang D, Yang CC, Wang Y. Harnessing social media for health information management. Electron Commer Res Appl. 2018;27:139–51.

4. Melchior C, Oliveira M. Health-related fake news on social media platforms: a systematic literature review. New Media Soc. 2021;24(6):1500–22.

5. Cinelli M, Quattrociocchi W, Galeazzi A, Valensise CM, Brugnoli E, Schmidt AL, et al. The COVID-19 social media infodemic. Sci Rep. 2020;10(1):16598.

6. Steinberg BJ, Hilton IV, Iida H, Samelson R. Oral health and dental care during pregnancy. Dent Clin North Am. 2013;57(2):195–210.

7. Baker B, Yang I. Social media as social support in pregnancy and the postpartum. Sex Reprod Healthc. 2018;17:31–4.

8. Jeihooni AK, Jamshidi H, Kashfi SM, Avand A, Khiyali Z. The effect of health education program based on health belief model on oral health behaviors in pregnant women of Fasa City, Fars Province, South of Iran. J Int Soc Prev Community Dent. 2017;7(6):336–43.

9. Chan KL, Chen M. Effects of social media and mobile health apps on pregnancy care: meta-analysis. JMIR Mhealth Uhealth. 2019;7(1):e11836.

10. Overdijkink SB, Velu AV, Rosman AN, van Beukering MD, Kok M, Steegers-Theunissen RP. The usability and effectiveness of mobile health technology-based lifestyle and medical intervention apps supporting health care during pregnancy: systematic review. JMIR Mhealth Uhealth. 2018;6(4):e109.

11. Vamos CA, Thompson EL, Avendano M, Daley EM, Quinonez RB, Boggess K. Oral health promotion interventions during pregnancy: a systematic review. Community Dent Oral Epidemiol. 2015;43(5):385–96.

12. Bates SB, Riedy CA. Changing knowledge and beliefs through an oral health pregnancy message. J Public Health Dent. 2012;72(2):104–11.

13. Piwek L, Joinson A. “What do they Snapchat about?” Patterns of use in time-limited instant messaging service. Comput Human Behav. 2016;54:358–67.

14. Cortis Mack C, Harding M, Davies N, Ward G. RECAPP-XPR: a smartphone application for presenting and recalling experimentally controlled stimuli over longer timescales. Behav Res Methods. 2019;51(4):1804–23.

15. Endeley RE. End-to-end encryption in messaging services and national security—case of WhatsApp messenger. J Inf Secur. 2018;9(1):95–9.

16. Sullivan LM. Essentials of biostatistics in public health. 2nd ed. Sudbury: Jones & Bartlett Learning; 2011.

17. Aboalshamat K, Abdulrahman S, Alowadi J, Al-Mutairy N, Fairak M, Alraithi N, et al. Endodontic treatment in pregnancy: knowledge, attitudes, and practices of dentists and interns in Jeddah, Saudi Arabia. Open Dent J. 2020;14(1):211–8.

18. American Dental Association. Pregnancy. 2021. https://www.ada.org/resources/research/science-and-research-institute/oral-health-topics/pregnancy. Accessed 14 Aug 2022.

19. American Pregnancy Association. Pregnancy and dental work. 2021. https://americanpregnancy.org/healthy-pregnancy/is-it-safe/dental-work-and-pregnancy/. Accessed 14 Aug 2022.

20. Aboalshamat K, Khayat A, Halwani R, Bitan A, Alansari R. The effects of gamification on antimicrobial resistance knowledge and its relationship to dentistry in Saudi Arabia: a randomized controlled trial. BMC Public Health. 2020;20(1):680.
