# Supplementary material for: The effects of social media (Snapchat) interventions on the knowledge of oral health during pregnancy among pregnant women in Saudi Arabia
Source: PLoS One. 2023 Feb 16;18(2):e0281908. doi: 10.1371/journal.pone.0281908 (PMC9934359; doi:10.1371/journal.pone.0281908)
Supplement: S2 File — (PDF) [file pone.0281908.s002.pdf]

SN	OSN	Group	Age	Marital status	Education	Family income	Do you visit the dentist regularly?	Are you afraid of visiting the dentist?	Children	First pregnancy ?	Pregnancey month ?
1	8	Study	35	Married	Higher education	From 5,000 to 15,000 SAR	Yes	No	2	No	9
2	10	Study	24	Married	Higher education	From 5,000 to 15,000 SAR	No	Yes	0	Yes	9
3	12	Study	38	Married	Bachelor degree	From 5,000 to 15,000 SAR	Only when needed	Yes	2	No	2
4	16	Study	27	Married	Bachelor degree	From 5,000 to 15,000 SAR	Yes	No	0	Yes	9
5	18	Study	24	Married	Bachelor degree	From 5,000 to 15,000 SAR	Only when needed	No	0	Yes	5
6	20	Study	27	Married	Bachelor degree	From 5,000 to 15,000 SAR	No	Yes	0	Yes	4
7	23	Study	25	Married	Bachelor degree	From 5,000 to 15,000 SAR	No	Yes	2	No	3
8	25	Study	35	Married	Bachelor degree	From 5,000 to 15,000 SAR	No	Yes	3	No	6
9	26	Study	21	Married	Bachelor degree	From 5,000 to 15,000 SAR	No	No	0	Yes	8
10	27	Study	39	Married	High school	From 5,000 to 15,000 SAR	No	No	6	No	2
11	28	Study	25	Married	Bachelor degree	From 5,000 to 15,000 SAR	No	No	0	Yes	9
12	32	Study	30	Married	Bachelor degree	From 5,000 to 15,000 SAR	No	No	0	Yes	6
13	33	Study	27	Married	Bachelor degree	From 5,000 to 15,000 SAR	Only when needed	No	0	Yes	6
14	35	Study	32	Married	High school	Less than 5,000 SAR	No	Yes	3	No	9
15	38	Study	24	Married	Bachelor degree	From 5,000 to 15,000 SAR	No	Yes	0	Yes	6
16	39	Study	27	Married	High school	Less than 5,000 SAR	No	Yes	1	Yes	9
17	41	Study	30	Married	Bachelor degree	Less than 5,000 SAR	No	No	2	No	6
18	42	Study	27	Married	Bachelor degree	From 5,000 to 15,000 SAR	Yes	No	1	No	3
19	47	Study	20	Married	High school	Less than 5,000 SAR	No	Yes	0	No	5
20	49	Study	20	Married	Bachelor degree	From 5,000 to 15,000 SAR	Yes	No	1	No	3
21	51	Study	35	Married	Bachelor degree	Less than 5,000 SAR	No	No	2	No	3
22	52	Study	33	Married	Bachelor degree	Less than 5,000 SAR	No	Yes	3	No	3
23	55	Study	33	Married	Bachelor degree	Less than 5,000 SAR	No	No	3	No	3
24	57	Study	27	Married	Higher education	From 5,000 to 15,000 SAR	Only when needed	Yes	1	No	6
25	58	Study	20	Married	Bachelor degree	From 5,000 to 15,000 SAR	No	No	0	Yes	7
26	59	Study	30	Married	Higher education	More than 15,000	No	No	0	Yes	6
27	62	Study	29	Married	Bachelor degree	From 5,000 to 15,000 SAR	No	No	1	No	6
28	64	Study	30	Married	High school	From 5,000 to 15,000 SAR	No	Yes	1	No	3
29	65	Study	29	Married	Bachelor degree	From 5,000 to 15,000 SAR	Only when needed	No	0	Yes	1
30	66	Study	27	Married	Bachelor degree	From 5,000 to 15,000 SAR	No	Yes	1	Yes	3
31	1	Control	27	Married	Bachelor degree	From 5,000 to 15,000 SAR	Only when needed	Yes	0	Yes	4
32	2	Control	35	Married	Higher education	Less than 5,000 SAR	No	Yes	3	No	5
33	3	Control	25	Married	Bachelor degree	From 5,000 to 15,000 SAR	No	No	0	Yes	5
34	4	Control	25	Married	Bachelor degree	Less than 5,000 SAR	No	No	0	Yes	3
35	5	Control	38	Married	Bachelor degree	Less than 5,000 SAR	No	Yes	5	No	6
36	6	Control	26	Married	High school	From 5,000 to 15,000 SAR	Only when needed	No	0	Yes	9
37	7	Control	25	Married	Bachelor degree	From 5,000 to 15,000 SAR	No	Yes	0	Yes	9
38	9	Control	26	Married	Bachelor degree	From 5,000 to 15,000 SAR	No	Yes	2	No	7
39	13	Control	25	Married	Bachelor degree	From 5,000 to 15,000 SAR	No	No	1	No	9
40	14	Control	28	Married	Bachelor degree	More than 15,000	Yes	No	3	No	4
41	15	Control	31	Married	Bachelor degree	More than 15,000	No	No	1	No	7
42	19	Control	32	Married	High school	Less than 5,000 SAR	No	No	4	No	1
43	21	Control	31	Married	Bachelor degree	More than 15,000	Yes	No	2	No	3
44	22	Control	23	Married	Bachelor degree	From 5,000 to 15,000 SAR	No	No	0	Yes	7
45	24	Control	30	Married	Bachelor degree	From 5,000 to 15,000 SAR	No	Yes	1	No	4
46	29	Control	32	Married	Bachelor degree	From 5,000 to 15,000 SAR	No	Yes	2	No	4
47	30	Control	30	Married	Bachelor degree	From 5,000 to 15,000 SAR	No	No	1	No	4
48	31	Control	28	Married	High school	From 5,000 to 15,000 SAR	Only when needed	No	1	No	2
49	34	Control	25	Married	Bachelor degree	Less than 5,000 SAR	Only when needed	No	1	No	6
50	36	Control	29	Married	Bachelor degree	From 5,000 to 15,000 SAR	No	No	3	No	9
51	37	Control	27	Married	Bachelor degree	From 5,000 to 15,000 SAR	No	No	0	Yes	8
52	40	Control	37	Married	Bachelor degree	From 5,000 to 15,000 SAR	No	Yes	3	No	5
53	43	Control	32	Married	Bachelor degree	From 5,000 to 15,000 SAR	Yes	No	0	Yes	8
54	44	Control	22	Married	High school	Less than 5,000 SAR	Only when needed	No	0	Yes	6
55	45	Control	34	Married	Bachelor degree	From 5,000 to 15,000 SAR	No	No	3	No	8
56	48	Control	25	Married	Bachelor degree	More than 15,000	No	Yes	1	No	7
57	50	Control	30	Married	Bachelor degree	From 5,000 to 15,000 SAR	No	Yes	0	Yes	5
58	53	Control	20	Married	High school	From 5,000 to 15,000 SAR	No	No	1	No	7
59	54	Control	33	Married	Higher education	From 5,000 to 15,000 SAR	Only when needed	No	1	No	4
60	56	Control	35	Married	High school	From 5,000 to 15,000 SAR	No	No	3	No	7
61	61	Control	27	Married	Bachelor degree	From 5,000 to 15,000 SAR	No	No	1	No	6
62	67	Control	30	Married	Bachelor degree	From 5,000 to 15,000 SAR	Only when needed	No	2	No	6
63	68	Control	33	Married	Bachelor degree	Less than 5,000 SAR	No	Yes	1	No	2

Info. Doctors	Info. Dentists	Info. Social media	Info. Internet	Info. TV	Info. Books	Info. Others	Average hours per day spent on social media	WhatsApp	Twitter	SnapChat	Instigram	Tik tok
Yes	Yes	No	No	No	No	No	4	Yes	Yes	Yes	Yes	No
Yes	Yes	Yes	Yes	No	Yes	No	4	Yes	Yes	Yes	Yes	No
No	Yes	Yes	Yes	No	No	No	5	Yes	No	Yes	Yes	No
No	Yes	Yes	Yes	No	No	Yes	4	Yes	Yes	Yes	Yes	Yes
Yes	Yes	Yes	No	No	No	No	4	Yes	Yes	Yes	Yes	Yes
No	Yes	Yes	Yes	No	No	No	4	Yes	Yes	Yes	Yes	No
No	No	Yes	Yes	Yes	Yes	Yes	4	Yes	No	Yes	Yes	No
Yes	Yes	Yes	Yes	No	No	No	3	No	No	Yes	Yes	No
No	Yes	Yes	Yes	No	No	Yes	5	Yes	Yes	Yes	Yes	Yes
No	No	Yes	Yes	No	No	Yes	3	Yes	Yes	Yes	Yes	Yes
No	Yes	Yes	Yes	No	No	No	4	Yes	Yes	Yes	Yes	Yes
No	Yes	Yes	Yes	No	No	Yes	5	Yes	No	Yes	Yes	No
Yes	Yes	Yes	Yes	No	No	Yes	5	Yes	Yes	Yes	Yes	Yes
Yes	Yes	No	Yes	No	No	No	5	Yes	No	Yes	Yes	Yes
Yes	Yes	Yes	Yes	No	No	No	7	Yes	Yes	Yes	Yes	No
Yes	Yes	Yes	Yes	Yes	Yes	No	2	Yes	No	Yes	No	No
Yes	Yes	Yes	Yes	No	No	Yes	10	Yes	Yes	Yes	Yes	No
Yes	Yes	No	Yes	No	No	No	4	Yes	Yes	Yes	No	No
No	Yes	No	Yes	No	No	No	3	Yes	No	No	No	No
Yes	Yes	Yes	Yes	No	Yes	Yes	6	Yes	Yes	Yes	No	Yes
No	Yes	Yes	Yes	No	No	No	3	Yes	Yes	Yes	Yes	No
Yes	Yes	Yes	Yes	No	No	No	4	Yes	Yes	Yes	Yes	Yes
Yes	Yes	Yes	Yes	Yes	No	No	3	Yes	No	Yes	Yes	Yes
No	Yes	No	Yes	No	No	No	7	Yes	Yes	Yes	Yes	Yes
No	No	Yes	Yes	No	No	Yes	8	Yes	Yes	Yes	Yes	Yes
No	Yes	Yes	Yes	No	No	No	3	Yes	Yes	Yes	Yes	Yes
Yes	No	Yes	Yes	No	No	No	6	Yes	No	Yes	Yes	No
Yes	Yes	No	Yes	Yes	No	No	7	Yes	Yes	Yes	Yes	Yes
No	Yes	Yes	Yes	No	No	Yes	3	Yes	No	Yes	Yes	No
Yes	Yes	Yes	Yes	No	No	Yes	9	Yes	Yes	Yes	Yes	Yes
Yes	Yes	Yes	Yes	No	No	No	5	Yes	No	Yes	Yes	Yes
Yes	Yes	Yes	Yes	No	No	No	3	No	No	No	Yes	Yes
No	Yes	Yes	Yes	No	No	No	5	No	Yes	Yes	Yes	No
Yes	Yes	Yes	Yes	No	No	No	5	Yes	Yes	Yes	Yes	Yes
No	Yes	Yes	Yes	No	Yes	No	4	Yes	No	Yes	Yes	No
No	Yes	No	No	No	No	No	12	No	No	Yes	No	No
Yes	No	Yes	Yes	No	No	No	5	Yes	Yes	Yes	Yes	Yes
Yes	Yes	Yes	Yes	No	No	Yes	12	Yes	Yes	Yes	Yes	Yes
No	Yes	Yes	Yes	Yes	Yes	Yes	5	Yes	No	Yes	Yes	No
Yes	Yes	Yes	Yes	No	No	No	2	Yes	Yes	Yes	Yes	No
No	Yes	Yes	No	No	No	No	4	Yes	No	Yes	Yes	No
No	Yes	Yes	Yes	No	Yes	No	6	Yes	Yes	Yes	Yes	Yes
Yes	Yes	Yes	Yes	Yes	No	No	2	Yes	Yes	No	No	No
No	No	Yes	Yes	No	No	No	10	Yes	Yes	Yes	No	Yes
Yes	Yes	Yes	Yes	Yes	No	No	2	Yes	Yes	Yes	Yes	No
Yes	Yes	Yes	Yes	No	No	No	2	Yes	No	Yes	Yes	Yes
No	Yes	No	Yes	Yes	Yes	No	3	Yes	No	Yes	Yes	No
No	Yes	No	Yes	No	No	No	4	Yes	No	Yes	Yes	No
No	Yes	Yes	Yes	No	No	No	6	Yes	Yes	Yes	No	Yes
Yes	Yes	Yes	Yes	Yes	Yes	Yes	3	Yes	Yes	Yes	Yes	No
No	Yes	No	No	No	No	No	8	Yes	Yes	Yes	Yes	No
No	Yes	Yes	Yes	No	No	Yes	5	Yes	No	Yes	Yes	Yes
Yes	Yes	Yes	No	No	No	No	2	Yes	No	Yes	Yes	No
No	Yes	Yes	Yes	Yes	No	No	6	Yes	No	Yes	Yes	No
Yes	Yes	No	Yes	No	No	No	2	Yes	Yes	Yes	Yes	No
Yes	Yes	Yes	Yes	No	No	No	2	Yes	Yes	Yes	No	No
Yes	Yes	Yes	Yes	Yes	Yes	Yes	4	Yes	No	Yes	Yes	No
No	Yes	No	No	No	No	No	8	Yes	No	Yes	Yes	Yes
No	Yes	Yes	Yes	No	No	No	8	Yes	Yes	Yes	No	Yes
Yes	Yes	Yes	Yes	No	No	Yes	6	Yes	No	Yes	Yes	Yes
Yes	Yes	Yes	Yes	Yes	Yes	Yes	2	Yes	No	Yes	Yes	Yes
No	No	Yes	Yes	No	No	No	6	Yes	No	Yes	Yes	Yes
No	No	Yes	Yes	No	No	No	2	Yes	No	Yes	Yes	Yes

Youtube	Telegram	Hours spend on Snapchat per day	Hours spend on WhatsApp per day	Do you often believe health information provided by social media influencers?	T1: Pregnant women cannot receive dental treatment at all, as it is unsafe.	T1: Pregnant women can receive emergency treatment such as root canal treatment or tooth extraction.	T1: The best time to receive emergency dental treatment is from the fourth month to the sixth month (second trimester)	T1: It is okay to receive non-emergency treatments, such as teeth whitening and cosmetic treatments in the last three months of pregnancy. (The last trimester)	T1: It is preferable that the appointments for visiting the dentist be short.
Yes	No	3	1	No	No	Yes	Yes	No	Yes
Yes	Yes	1	3	Neutrak	No	No	Yes	No	Yes
No	No	3	2	No	No	Yes	Yes	Yes	I do not know
No	Yes	3	1	No	No	Yes	Yes	Yes	Yes
Yes	Yes	1	2	Neutrak	No	I do not know	Yes	I do not know	I do not know
Yes	No	2	1	Neutrak	I do not know	I do not know	I do not know	I do not know	I do not know
Yes	No	2	1	Neutrak	I do not know	I do not know	Yes	I do not know	No
No	No	3	1	Neutrak	I do not know	Yes	Yes	No	I do not know
Yes	Yes	3	3	Neutrak	I do not know	Yes	Yes	No	I do not know
Yes	Yes	1	0	No	Yes	Yes	Yes	No	Yes
Yes	Yes	1	1	Yes	No	Yes	Yes	I do not know	No
Yes	Yes	2	1	Neutrak	No	Yes	I do not know	I do not know	No
Yes	Yes	2	1	Neutrak	No	I do not know	Yes	No	I do not know
No	No	4	4	No	Yes	No	I do not know	No	Yes
No	No	4	2	Neutrak	Yes	No	I do not know	No	Yes
Yes	No	1	0	No	No	Yes	Yes	Yes	Yes
Yes	Yes	4	3	Neutrak	No	Yes	Yes	I do not know	Yes
Yes	No	2	2	Neutrak	Yes	I do not know	No	No	Yes
Yes	Yes	0	6	No	No	Yes	Yes	No	Yes
No	Yes	3	1	Neutrak	No	Yes	Yes	No	Yes
No	No	1	1	Neutrak	No	Yes	Yes	I do not know	No
No	Yes	6	3	No	I do not know	Yes	I do not know	I do not know	No
Yes	Yes	1	1	Neutrak	No	No	Yes	Yes	No
No	No	2	2	Neutrak	No	Yes	Yes	No	No
Yes	No	4	4	Neutrak	Yes	No	I do not know	I do not know	Yes
Yes	Yes	2	1	Neutrak	No	Yes	No	I do not know	No
Yes	No	3	1	Neutrak	No	I do not know	I do not know	I do not know	I do not know
Yes	Yes	8	2	No	I do not know	I do not know	I do not know	No	I do not know
No	No	1	1	Neutrak	No	Yes	Yes	No	No
Yes	Yes	2	4	Neutrak	Yes	I do not know	Yes	No	Yes
Yes	Yes	2	1	Neutrak	No	Yes	Yes	I do not know	Yes
Yes	No	1	1	Neutrak	No	Yes	Yes	No	I do not know
No	No	1	1	Neutrak	No	I do not know	Yes	I do not know	I do not know
Yes	Yes	2	1	Yes	No	Yes	I do not know	I do not know	I do not know
Yes	No	1	3	Yes	Yes	Yes	Yes	Yes	Yes
No	No	5	2	Yes	I do not know	No	I do not know	No	Yes
No	No	3	2	Neutrak	No	Yes	Yes	I do not know	Yes
No	No	5	8	Neutrak	No	Yes	I do not know	No	I do not know
Yes	No	2	1	Neutrak	I do not know	I do not know	I do not know	I do not know	Yes
No	No	1	2	Neutrak	No	Yes	Yes	Yes	Yes
Yes	No	3	1	Neutrak	No	Yes	Yes	I do not know	Yes
Yes	Yes	3	2	Yes	No	Yes	Yes	Yes	No
Yes	Yes	1	2	Yes	No	Yes	Yes	No	No
Yes	No	5	1	Neutrak	Yes	No	No	No	No
Yes	No	1	1	Neutrak	No	Yes	Yes	Yes	Yes
No	No	3	2	Neutrak	No	Yes	Yes	No	Yes
Yes	Yes	1	1	No	No	Yes	Yes	I do not know	No
Yes	No	6	6	Neutrak	Yes	I do not know	Yes	I do not know	Yes
No	No	2	1	Neutrak	I do not know	Yes	Yes	I do not know	Yes
Yes	Yes	2	1	Neutrak	No	Yes	I do not know	No	Yes
No	No	3	1	Neutrak	No	I do not know	No	No	Yes
Yes	Yes	2	0	Neutrak	No	Yes	No	Yes	No
No	No	1	1	Neutrak	No	Yes	I do not know	Yes	Yes
Yes	Yes	4	4	No	No	No	I do not know	I do not know	I do not know
Yes	Yes	1	1	No	Yes	No	Yes	No	No
Yes	Yes	1	1	No	No	Yes	No	I do not know	I do not know
Yes	Yes	1	1	Yes	Yes	No	I do not know	No	Yes
Yes	No	2	2	Neutrak	I do not know	I do not know	I do not know	I do not know	No
No	No	2	1	No	No	I do not know	I do not know	I do not know	I do not know
Yes	Yes	4	10	Yes	No	Yes	Yes	No	Yes
Yes	Yes	1	1	No	No	Yes	I do not know	I do not know	No
No	No	2	3	Yes	No	Yes	Yes	Yes	I do not know
Yes	No	1	1	Neutrak	I do not know	I do not know	I do not know	I do not know	I do not know

T1: Endo treatment is important to avoid complications such as inflammation and swelling that can affect the airway	T1: Most of the medicinal substances used to treat nerve pain are not safe for pregnant women	T1: The best position for a pregnant woman in the dental chair is a semi-reclined position with a pillow under the patient's right side.	T1: X-rays are not safe for pregnant women or infants even with necessary precautions.	T1: When a pregnant woman is exposed to X-rays, she should wear a lead urpon and a thyroid collar to protect the thyroid gland.	T1: X-rays are essential in various dental procedures, especially in emergency situations.	T1: Routine X-rays, usually taken during annual checkups, are best taken during pregnancy.	T1: Is it forbidden for pregnant women to use all types of dental local anesthetics	T1: The most common antibiotics, such as amoxicillin, clindamycin, metronidazole, and penicillin (High Musk, Amoxil), are safe for pregnant patients.
Yes	I do not know	I do not know	Yes	I do not know	No	No	No	I do not know
Yes	Yes	Yes	No	Yes	Yes	Yes	No	I do not know
No	I do not know	I do not know	Yes	No	I do not know	I do not know	No	I do not know
Yes	I do not know	I do not know	No	Yes	Yes	No	No	Yes
I do not know	I do not know	I do not know	I do not know	Yes	Yes	I do not know	I do not know	I do not know
I do not know	I do not know	I do not know	Yes	Yes	Yes	I do not know	I do not know	I do not know
Yes	I do not know	I do not know	I do not know	I do not know	I do not know	I do not know	I do not know	I do not know
Yes	I do not know	I do not know	Yes	Yes	Yes	No	I do not know	I do not know
No	Yes	Yes	Yes	No	Yes	No	I do not know	I do not know
Yes	Yes	I do not know	Yes	Yes	Yes	No	I do not know	I do not know
Yes	I do not know	No	Yes	Yes	Yes	I do not know	No	No
Yes	No	Yes	I do not know	Yes	I do not know	I do not know	No	No
I do not know	I do not know	I do not know	Yes	I do not know	I do not know	No	I do not know	No
Yes	Yes	No	Yes	Yes	No	No	Yes	No
Yes	Yes	Yes	I do not know	I do not know	I do not know	I do not know	Yes	No
No	No	Yes	No	Yes	Yes	No	Yes	Yes
Yes	I do not know	Yes	Yes	Yes	I do not know	No	No	I do not know
Yes	Yes	Yes	I do not know	No	Yes	I do not know	I do not know	No
I do not know	I do not know	I do not know	Yes	I do not know	I do not know	No	No	I do not know
Yes	Yes	Yes	Yes	Yes	Yes	No	Yes	No
Yes	I do not know	I do not know	No	Yes	Yes	No	No	Yes
Yes	Yes	I do not know	Yes	No	No	No	I do not know	I do not know
Yes	I do not know	I do not know	I do not know	Yes	I do not know	Yes	I do not know	I do not know
I do not know	Yes	I do not know	Yes	I do not know	I do not know	No	I do not know	I do not know
I do not know	Yes	I do not know	Yes	I do not know	Yes	I do not know	I do not know	I do not know
Yes	No	Yes	Yes	Yes	Yes	No	No	I do not know
I do not know	I do not know	I do not know	Yes	I do not know	I do not know	I do not know	I do not know	I do not know
I do not know	I do not know	I do not know	Yes	Yes	Yes	I do not know	I do not know	No
Yes	Yes	I do not know	Yes	Yes	Yes	Yes	Yes	I do not know
No	Yes	Yes	Yes	Yes	Yes	No	Yes	No
Yes	I do not know	I do not know	I do not know	Yes	I do not know	I do not know	No	I do not know
Yes	No	I do not know	Yes	Yes	Yes	Yes	No	Yes
Yes	I do not know	I do not know	Yes	Yes	Yes	I do not know	I do not know	I do not know
Yes	No	I do not know	I do not know	I do not know	I do not know	I do not know	I do not know	I do not know
Yes	Yes	Yes	Yes	Yes	I do not know	I do not know	No	I do not know
Yes	Yes	I do not know	Yes	I do not know	I do not know	I do not know	Yes	Yes
Yes	I do not know	Yes	I do not know	I do not know	I do not know	I do not know	I do not know	I do not know
I do not know	I do not know	I do not know	Yes	I do not know	I do not know	No	I do not know	I do not know
Yes	I do not know	I do not know	I do not know	I do not know	Yes	I do not know	I do not know	I do not know
Yes	No	Yes	No	Yes	Yes	No	No	No
Yes	No	No	No	No	Yes	No	No	Yes
Yes	No	Yes	No	Yes	No	No	No	Yes
I do not know	I do not know	I do not know	Yes	I do not know	I do not know	I do not know	I do not know	I do not know
No	Yes	No	Yes	Yes	No	No	No	No
No	Yes	Yes	Yes	Yes	Yes	No	Yes	No
Yes	No	I do not know	I do not know	I do not know	I do not know	I do not know	No	I do not know
I do not know	Yes	I do not know	No	Yes	Yes	Yes	No	No
I do not know	I do not know	I do not know	Yes	I do not know	I do not know	I do not know	I do not know	I do not know
Yes	Yes	I do not know	Yes	Yes	I do not know	I do not know	I do not know	No
Yes	I do not know	I do not know	Yes	Yes	Yes	I do not know	No	I do not know
Yes	No	Yes	I do not know	No	I do not know	I do not know	No	I do not know
Yes	No	Yes	No	Yes	Yes	I do not know	No	I do not know
Yes	No	I do not know	Yes	Yes	I do not know	I do not know	No	No
I do not know	Yes	Yes	Yes	I do not know	Yes	Yes	Yes	No
Yes	Yes	Yes	Yes	Yes	Yes	Yes	Yes	No
Yes	No	I do not know	I do not know	Yes	No	No	No	Yes
Yes	Yes	I do not know	Yes	I do not know	I do not know	I do not know	Yes	I do not know
Yes	I do not know	I do not know	Yes	No	I do not know	I do not know	I do not know	I do not know
Yes	Yes	I do not know	Yes	I do not know	I do not know	I do not know	Yes	No
Yes	I do not know	I do not know	Yes	I do not know	Yes	I do not know	No	No
Yes	I do not know	I do not know	I do not know	I do not know	I do not know	I do not know	I do not know	I do not know
Yes	No	Yes	Yes	I do not know	I do not know	I do not know	No	No
I do not know	I do not know	I do not know	I do not know	I do not know	I do not know	I do not know	I do not know	I do not know

T1: Acetaminophen (paracetamol) such as Panadol and Vivadol: the safest analgesic for pregnant and breast-feeding patients.	T1: Non-steroidal anti-inflammatory drugs such as ibuprofen: safe during pregnancy (such as ibuprofen).	T1: Gingivitis usually increases during pregnancy from the fourth to the sixth month (second trimester); Which is known as: redness, swelling, bleeding, pain, difficulty chewing, movement of the teeth	T1: Preventive dental cleanings and annual exams during pregnancy are safe	T1: To reduce gum disease: brush your teeth twice with a soft brush and fluoride toothpaste and use dental floss	T1: Do not gargle with salt water	T2: Pregnant women cannot receive dental treatment at all, as it is unsafe.	T2: Pregnant women can receive emergency treatment such as root canal treatment or tooth extraction.	T2: The best time to receive emergency dental treatment is from the fourth month to the sixth month (second trimester)
Yes	No	No	Yes	Yes	No	No	Yes	Yes
Yes	I do not know	Yes	Yes	Yes	No	No	Yes	Yes
Yes	I do not know	Yes	I do not know	I do not know	No	No	Yes	Yes
Yes	No	Yes	Yes	Yes	No	No	Yes	Yes
I do not know	I do not know	Yes	Yes	Yes	No	No	Yes	Yes
Yes	I do not know	I do not know	Yes	Yes	No	No	Yes	No
I do not know	I do not know	Yes	Yes	Yes	No	No	Yes	Yes
Yes	No	Yes	I do not know	Yes	No	No	Yes	Yes
Yes	I do not know	Yes	Yes	Yes	No	No	Yes	Yes
No	No	Yes	Yes	Yes	No	I do not know	I do not know	I do not know
Yes	No	Yes	Yes	Yes	No	No	No	Yes
Yes	I do not know	I do not know	Yes	Yes	No	No	Yes	Yes
Yes	No	I do not know	No	Yes	No	No	Yes	Yes
Yes	I do not know	Yes	I do not know	Yes	I do not know	I do not know	Yes	I do not know
Yes	I do not know	Yes	I do not know	I do not know	I do not know	No	Yes	Yes
No	No	Yes	No	I do not know	No	No	Yes	Yes
Yes	I do not know	Yes	Yes	Yes	No	No	Yes	Yes
Yes	I do not know	I do not know	I do not know	Yes	No	No	Yes	Yes
Yes	I do not know	No	Yes	Yes	No	No	Yes	Yes
Yes	Yes	Yes	Yes	Yes	No	Yes	Yes	Yes
Yes	No	Yes	I do not know	Yes	No	No	Yes	Yes
No	No	No	Yes	Yes	No	No	Yes	Yes
Yes	I do not know	I do not know	Yes	Yes	No	No	Yes	Yes
Yes	No	I do not know	Yes	Yes	No	No	Yes	Yes
Yes	I do not know	No	I do not know	Yes	I do not know	No	Yes	No
Yes	I do not know	Yes	Yes	Yes	No	No	Yes	Yes
Yes	I do not know	I do not know	I do not know	Yes	I do not know	No	Yes	Yes
I do not know	No	Yes	Yes	Yes	No	No	Yes	I do not know
I do not know	I do not know	Yes	No	I do not know	No	No	Yes	Yes
Yes	No	I do not know	No	Yes	No	No	Yes	Yes
Yes	No	Yes	Yes	Yes	No	No	Yes	Yes
Yes	No	Yes	Yes	Yes	No	No	Yes	Yes
Yes	No	Yes	Yes	Yes	I do not know	No	Yes	Yes
Yes	No	Yes	Yes	Yes	No	No	Yes	Yes
Yes	No	Yes	Yes	Yes	I do not know	No	Yes	Yes
Yes	No	Yes	Yes	Yes	No	No	Yes	Yes
Yes	I do not know	No	Yes	Yes	No	No	Yes	Yes
Yes	No	Yes	Yes	Yes	No	No	Yes	Yes
No	I do not know	Yes	Yes	Yes	I do not know	No	Yes	Yes
Yes	No	No	Yes	Yes	No	No	Yes	No
Yes	Yes	Yes	Yes	Yes	No	No	Yes	Yes
Yes	No	Yes	Yes	Yes	No	No	Yes	No
I do not know	No	Yes	I do not know	Yes	No	No	Yes	Yes
Yes	No	Yes	Yes	No	No	No	Yes	Yes
Yes	Yes	Yes	Yes	Yes	No	No	Yes	Yes
Yes	I do not know	I do not know	I do not know	I do not know	No	No	Yes	Yes
Yes	No	No	Yes	Yes	No	No	Yes	Yes
Yes	I do not know	I do not know	Yes	Yes	I do not know	Yes	Yes	Yes
Yes	No	Yes	I do not know	Yes	No	No	Yes	Yes
Yes	No	Yes	Yes	Yes	No	No	Yes	Yes
Yes	I do not know	I do not know	Yes	Yes	No	No	Yes	Yes
Yes	No	Yes	Yes	Yes	No	No	Yes	Yes
Yes	Yes	I do not know	Yes	Yes	No	No	Yes	Yes
Yes	No	Yes	I do not know	No	No	No	No	No
Yes	No	Yes	Yes	Yes	No	No	Yes	Yes
Yes	I do not know	Yes	Yes	Yes	I do not know	No	Yes	No
Yes	Yes	I do not know	I do not know	Yes	No	No	Yes	Yes
Yes	I do not know	Yes	I do not know	Yes	No	No	Yes	No
Yes	I do not know	Yes	I do not know	Yes	No	No	Yes	No
Yes	No	Yes	I do not know	Yes	No	No	Yes	Yes
Yes	No	Yes	Yes	Yes	No	No	Yes	No
Yes	No	Yes	Yes	Yes	No	No	Yes	Yes
Yes	I do not know	I do not know	I do not know	Yes	No	No	Yes	Yes

T2: It is okay to receive non-emergency treatments, such as teeth whitening and cosmetic treatments in the last three months of pregnancy. (The last trimester)	T2: It is preferable that the appointments for visiting the dentist be short.	T2: Endo treatment is important to avoid complications such as inflammation and swelling that can affect the airway	T2: Most of the medicinal substances used to treat nerve pain are not safe for pregnant women	T2: The best position for a pregnant woman in the dental chair is a semi-reclined position with a pillow under the patient's right side.	T2: X-rays are not safe for pregnant women or infants even with necessary precautions.	T2: When a pregnant woman is exposed to X-rays, she should wear a lead urpon and a thyroid collar to protect the thyroid gland.	T2: X-rays are essential in various dental procedures, especially in emergency situations.	T2: Routine X-rays, usually taken during annual checkups, are best taken during pregnancy.
No	Yes	Yes	Yes	Yes	No	Yes	No	No
No	Yes	Yes	Yes	Yes	No	Yes	No	Yes
No	Yes	Yes	No	I do not know	Yes	Yes	Yes	Yes
No	Yes	Yes	No	Yes	No	Yes	Yes	No
No	Yes	Yes	No	Yes	No	Yes	Yes	No
No	No	Yes	No	Yes	No	Yes	Yes	No
Yes	No	Yes	No	Yes	Yes	I do not know	I do not know	No
Yes	Yes	Yes	No	Yes	No	Yes	Yes	Yes
No	No	Yes	No	Yes	I do not know	Yes	Yes	No
No	Yes	Yes	Yes	I do not know	Yes	Yes	Yes	I do not know
Yes	Yes	I do not know	I do not know	Yes	Yes	I do not know	No	I do not know
No	Yes	Yes	No	Yes	No	Yes	No	No
No	Yes	Yes	No	Yes	No	Yes	Yes	I do not know
Yes	Yes	Yes	Yes	No	Yes	Yes	No	I do not know
No	Yes	Yes	No	Yes	No	Yes	Yes	No
Yes	Yes	No	No	Yes	No	Yes	Yes	No
No	Yes	No	No	Yes	No	Yes	No	I do not know
No	Yes	Yes	No	Yes	No	Yes	Yes	No
Yes	Yes	Yes	No	Yes	No	Yes	Yes	No
Yes	No	Yes	No	Yes	Yes	Yes	Yes	Yes
No	No	Yes	No	Yes	No	Yes	Yes	No
Yes	Yes	Yes	Yes	Yes	No	Yes	Yes	Yes
No	Yes	Yes	No	Yes	No	Yes	No	Yes
No	Yes	Yes	No	Yes	No	Yes	No	No
No	Yes	Yes	No	Yes	No	Yes	No	I do not know
No	Yes	Yes	I do not know	Yes	No	Yes	Yes	No
No	Yes	Yes	No	Yes	No	Yes	No	No
I do not know	No	I do not know	I do not know	I do not know	Yes	Yes	Yes	I do not know
Yes	No	Yes	Yes	Yes	Yes	Yes	I do not know	Yes
No	Yes	Yes	No	Yes	No	Yes	Yes	Yes
I do not know	Yes	Yes	No	Yes	Yes	Yes	I do not know	I do not know
No	Yes	Yes	No	I do not know	No	Yes	Yes	No
No	No	Yes	Yes	Yes	Yes	Yes	Yes	No
No	Yes	Yes	No	Yes	No	Yes	No	No
No	Yes	Yes	Yes	Yes	Yes	Yes	No	No
No	Yes	Yes	No	Yes	No	Yes	No	No
No	Yes	Yes	No	Yes	No	No	I do not know	I do not know
No	Yes	Yes	No	I do not know	No	Yes	Yes	No
No	Yes	Yes	No	Yes	No	No	I do not know	No
Yes	Yes	Yes	No	Yes	No	Yes	Yes	No
No	Yes	Yes	No	Yes	No	Yes	No	Yes
Yes	Yes	Yes	No	Yes	No	Yes	No	No
No	I do not know	Yes	No	Yes	No	Yes	I do not know	No
No	Yes	Yes	No	No	No	Yes	Yes	No
No	Yes	Yes	No	Yes	No	Yes	No	No
No	Yes	Yes	No	I do not know	No	Yes	Yes	No
No	Yes	Yes	No	Yes	No	Yes	Yes	No
No	Yes	I do not know	No	Yes	Yes	Yes	I do not know	Yes
No	No	Yes	No	Yes	Yes	Yes	Yes	Yes
No	Yes	Yes	No	Yes	No	Yes	Yes	No
No	No	Yes	No	Yes	No	I do not know	No	No
Yes	No	Yes	No	Yes	No	Yes	Yes	Yes
No	Yes	Yes	No	Yes	No	Yes	Yes	No
Yes	No	I do not know	I do not know	Yes	I do not know	I do not know	I do not know	I do not know
No	Yes	No	Yes	Yes	Yes	Yes	No	No
No	Yes	Yes	No	Yes	No	Yes	I do not know	No
No	Yes	Yes	No	Yes	No	Yes	Yes	No
No	Yes	Yes	No	Yes	No	Yes	No	No
No	Yes	Yes	No	Yes	No	Yes	Yes	No
No	Yes	Yes	No	Yes	Yes	Yes	Yes	Yes
No	Yes	Yes	No	I do not know	No	Yes	No	No
No	Yes	Yes	No	Yes	No	Yes	Yes	Yes
No	Yes	Yes	Yes	Yes	No	Yes	Yes	No

T2: Is it forbidden for pregnant women to use all types of dental local anesthetics	T2: The most common antibiotics, such as amoxicillin, clindamycin, metronidazole, and penicillin (High Musk, Amoxil), are safe for pregnant patients.	T2: Acetaminophen (paracetamol) such as Panadol and Vivadol: the safest analgesic for pregnant and breast-feeding patients.	T2: Non-steroidal anti-inflammatory drugs such as ibuprofen: safe during pregnancy (such as ibuprofen).	T2: Gingivitis usually increases during pregnancy from the fourth to the sixth month (second trimester); Which is known as: redness, swelling, bleeding, pain, difficulty chewing, movement of the teeth	T2: Preventive dental cleanings and annual exams during pregnancy are safe	T2: To reduce gum disease: brush your teeth twice with a soft brush and fluoride toothpaste and use dental floss	T2: Do not gargle with salt water	I am generally fully satisfied with receving the health information via Snapchat experience.
No	No	Yes	No	Yes	Yes	Yes	No	Strongly agree
No	Yes	Yes	No	Yes	Yes	Yes	No	Strongly agree
No	Yes	Yes	No	Yes	I do not know	Yes	No	Strongly agree
No	Yes	Yes	No	Yes	Yes	Yes	No	Strongly agree
No	No	Yes	No	Yes	Yes	Yes	No	Strongly agree
No	Yes	Yes	No	Yes	Yes	Yes	No	Strongly agree
No	No	Yes	No	Yes	Yes	Yes	No	Strongly disagree
No	Yes	Yes	No	Yes	Yes	Yes	No	Strongly agree
No	No	Yes	Yes	Yes	Yes	Yes	No	Strongly disagree
I do not know	No	No	No	Yes	Yes	Yes	No	Neutral
I do not know	I do not know	Yes	No	I do not know	Yes	Yes	No	Strongly disagree
No	No	Yes	No	Yes	Yes	Yes	No	Agree
No	No	Yes	No	Yes	Yes	Yes	No	Agree
No	I do not know	Yes	Yes	Yes	I do not know	Yes	No	Agree
No	No	Yes	No	Yes	Yes	Yes	No	Agree
No	No	Yes	No	Yes	Yes	Yes	No	Strongly disagree
No	Yes	Yes	No	Yes	Yes	Yes	No	Agree
No	Yes	Yes	No	Yes	Yes	Yes	No	Strongly agree
No	No	Yes	No	Yes	No	Yes	Yes	Strongly disagree
Yes	No	Yes	Yes	Yes	Yes	Yes	No	Strongly disagree
No	Yes	Yes	No	Yes	Yes	Yes	No	Strongly agree
No	No	Yes	No	Yes	Yes	Yes	No	Agree
No	Yes	Yes	No	Yes	Yes	Yes	No	Strongly disagree
No	No	Yes	Yes	Yes	Yes	Yes	No	Agree
No	Yes	Yes	No	Yes	Yes	Yes	No	Strongly agree
No	I do not know	Yes	No	Yes	Yes	Yes	No	Agree
No	Yes	Yes	No	Yes	Yes	Yes	No	Agree
I do not know	I do not know	I do not know	I do not know	I do not know	Yes	Yes	No	Strongly disagree
I do not know	No	Yes	No	Yes	Yes	Yes	No	Strongly disagree
No	No	Yes	No	Yes	Yes	Yes	No	Strongly disagree
No	Yes	Yes	No	Yes	Yes	Yes	No	
No	Yes	Yes	Yes	Yes	Yes	Yes	No	
No	No	Yes	No	Yes	Yes	Yes	No	
No	Yes	Yes	No	Yes	Yes	Yes	No	
No	No	Yes	No	Yes	Yes	Yes	No	
No	Yes	Yes	Yes	Yes	Yes	Yes	No	
No	I do not know	Yes	No	I do not know	I do not know	Yes	No	
No	Yes	Yes	No	Yes	Yes	Yes	No	
No	Yes	No	No	Yes	Yes	Yes	No	
No	Yes	Yes	No	Yes	Yes	Yes	No	
No	Yes	Yes	No	Yes	Yes	Yes	No	
No	Yes	Yes	No	Yes	Yes	Yes	No	
Yes	Yes	Yes	No	Yes	Yes	Yes	No	
No	No	Yes	No	Yes	Yes	Yes	No	
No	Yes	Yes	Yes	Yes	Yes	Yes	No	
Yes	Yes	Yes	I do not know	Yes	Yes	Yes	No	
No	Yes	Yes	No	Yes	Yes	Yes	No	
I do not know	Yes	Yes	No	Yes	Yes	Yes	No	
No	Yes	No	No	Yes	Yes	Yes	No	
No	Yes	Yes	No	Yes	Yes	Yes	No	
No	No	Yes	Yes	Yes	Yes	Yes	No	
No	Yes	Yes	No	Yes	Yes	Yes	No	
No	Yes	Yes	No	No	Yes	Yes	No	
Yes	I do not know	Yes	I do not know	Yes	Yes	Yes	I do not know	
No	No	Yes	No	Yes	No	Yes	No	
No	Yes	Yes	No	Yes	I do not know	Yes	No	
No	Yes	Yes	Yes	Yes	Yes	Yes	No	
No	Yes	Yes	Yes	Yes	Yes	Yes	No	
No	Yes	Yes	No	Yes	Yes	Yes	No	
No	No	Yes	No	Yes	Yes	Yes	No	
No	Yes	Yes	No	Yes	Yes	Yes	No	
No	Yes	Yes	No	Yes	Yes	Yes	No	
Yes	No	Yes	No	Yes	No	Yes	No	

Receiving oral health information via Snapchat was helpful to me	Receiving oral health information via Snapchat was enjoyable.	Receiving oral health information via Snapchat was innovative.	Accessing the Snapchat application easy for you.	The duration of the snapchat promotional program was appropriate.	The information was easy to understand.	I advise pregnant women to recieve oral health information through snapchat	I will follow Snapchat accounts that transmit useful oral health information.
Strongly agree	Agree	Agree	Strongly agree	Agree	Strongly agree	Agree	Strongly agree
Strongly agree	Strongly agree	Strongly agree	Neutral	Strongly agree	Strongly agree	Agree	Agree
Strongly agree	Strongly agree	Strongly agree	Strongly agree	Strongly agree	Strongly agree	Strongly agree	Strongly agree
Strongly agree	Agree	Agree	Strongly agree	Strongly agree	Agree	Strongly agree	Agree
Strongly agree	Strongly agree	Strongly agree	Strongly agree	Strongly agree	Strongly agree	Agree	Agree
Strongly agree	Strongly agree	Strongly agree	Strongly agree	Strongly agree	Strongly agree	Strongly agree	Strongly agree
Strongly disagree	Strongly disagree	Strongly disagree	Strongly disagree	Disagree	Strongly disagree	Strongly disagree	Neutral
Agree	Strongly agree	Strongly agree	Strongly agree	Strongly agree	Strongly agree	Agree	Agree
Strongly disagree	Strongly disagree	Strongly disagree	Strongly disagree	Strongly disagree	Strongly disagree	Strongly disagree	Strongly disagree
Agree	Agree	Agree	Agree	Strongly disagree	Agree	Strongly disagree	Strongly disagree
Strongly disagree	Strongly disagree	Strongly disagree	Strongly disagree	Strongly disagree	Strongly disagree	Strongly disagree	Strongly disagree
Neutral	Neutral	Agree	Agree	Neutral	Agree	Agree	Agree
Strongly agree	Agree	Strongly agree	Agree	Agree	Agree	Agree	Agree
Agree	Agree	Disagree	Agree	Neutral	Disagree	Disagree	Disagree
Agree	Agree	Agree	Agree	Agree	Agree	Agree	Agree
Strongly disagree	Strongly disagree	Strongly disagree	Strongly disagree	Strongly disagree	Strongly disagree	Strongly disagree	Strongly disagree
Agree	Agree	Agree	Agree	Agree	Agree	Agree	Agree
Strongly agree	Strongly agree	Strongly agree	Strongly agree	Strongly agree	Strongly agree	Strongly agree	Strongly agree
Strongly disagree	Strongly disagree	Strongly disagree	Strongly disagree	Strongly disagree	Strongly disagree	Strongly disagree	Strongly disagree
Strongly disagree	Strongly disagree	Strongly disagree	Strongly disagree	Strongly disagree	Strongly disagree	Strongly disagree	Strongly disagree
Strongly agree	Strongly agree	Strongly agree	Strongly agree	Strongly agree	Strongly agree	Strongly agree	Strongly agree
Agree	Agree	Agree	Agree	Agree	Agree	Agree	Agree
Strongly disagree	Strongly disagree	Strongly disagree	Strongly disagree	Strongly disagree	Strongly disagree	Agree	Agree
Agree	Agree	Neutral	Agree	Agree	Agree	Agree	Neutral
Strongly agree	Strongly agree	Agree	Strongly agree	Strongly agree	Strongly agree	Agree	Neutral
Disagree	Neutral	Disagree	Neutral	Neutral	Neutral	Disagree	Neutral
Strongly agree	Agree	Agree	Strongly agree	Agree	Agree	Agree	Agree
Neutral	Neutral	Strongly disagree	Strongly disagree	Strongly disagree	Strongly disagree	Strongly disagree	Strongly disagree
Strongly disagree	Strongly disagree	Strongly disagree	Strongly disagree	Strongly disagree	Strongly disagree	Strongly disagree	Strongly disagree
Strongly disagree	Strongly disagree	Strongly disagree	Strongly disagree	Strongly disagree	Strongly disagree	Strongly disagree	Strongly disagree

Knowing that the oral health information were revied scentifically was important to me.	I am generally fully satisfied with receving the health information via whatsApp experience.	Receiving oral health information via WhatsApp was helpful to me	Receiving oral health information via WhatsApp was enjoyable.	Receiving oral health information via WhatsApp was innovative.	Accessing the WhatsApp application easy for you.	The duration of the WhatsApp promotional program was appropriate.	The information was easy to understand.
Strongly agree							
Strongly agree							
Strongly agree							
Strongly agree							
Strongly agree							
Strongly agree							
Strongly disagree							
Agree							
Strongly disagree							
Neutral							
Strongly disagree							
Agree							
Agree							
Disagree							
Agree							
Strongly disagree							
Agree							
Strongly agree							
Strongly disagree							
Strongly disagree							
Strongly agree							
Agree							
Agree							
Agree							
Agree							
Disagree							
Agree							
Strongly disagree							
Strongly disagree							
Strongly disagree							
	Strongly agree	Strongly agree	Strongly agree	Strongly agree	Strongly agree	Strongly agree	Strongly agree
	Strongly agree	Strongly agree	Strongly agree	Strongly agree	Strongly agree	Strongly agree	Strongly agree
	Strongly disagree	Disagree	Strongly disagree	Strongly disagree	Neutral	Agree	Agree
	Strongly agree	Strongly agree	Strongly agree	Strongly agree	Strongly agree	Strongly agree	Strongly agree
	Strongly disagree	Strongly disagree	Strongly disagree	Strongly disagree	Strongly disagree	Strongly disagree	Strongly disagree
	Agree	Agree	Neutral	Neutral	Agree	Agree	Agree
	Strongly agree	Strongly agree	Strongly agree	Strongly agree	Strongly agree	Strongly agree	Strongly agree
	Strongly agree	Strongly agree	Strongly agree	Strongly agree	Strongly agree	Strongly agree	Strongly agree
	Strongly agree	Strongly agree	Agree	Agree	Strongly agree	Agree	Strongly agree
	Strongly agree	Strongly agree	Strongly agree	Agree	Strongly agree	Strongly agree	Strongly agree
	Strongly disagree	Strongly disagree	Strongly disagree	Strongly disagree	Strongly disagree	Strongly disagree	Strongly disagree
	Agree	Agree	Agree	Agree	Agree	Agree	Agree
	Strongly agree	Strongly agree	Strongly agree	Strongly agree	Strongly agree	Strongly agree	Strongly agree
	Strongly disagree	Strongly disagree	Strongly disagree	Strongly disagree	Strongly disagree	Strongly disagree	Strongly disagree
	Agree	Agree	Agree	Agree	Agree	Agree	Agree
	Agree	Agree	Agree	Agree	Agree	Agree	Agree
	Strongly agree	Strongly agree	Strongly agree	Strongly agree	Strongly agree	Strongly agree	Strongly agree
	Agree	Agree	Agree	Agree	Agree	Agree	Agree
	Agree	Strongly agree	Strongly agree	Strongly agree	Strongly agree	Strongly agree	Strongly agree
	Agree	Agree	Neutral	Neutral	Agree	Agree	Agree
	Strongly agree	Agree	Agree	Strongly agree	Strongly agree	Strongly agree	Strongly agree
	Strongly disagree	Strongly disagree	Strongly disagree	Strongly disagree	Strongly disagree	Strongly disagree	Strongly disagree
	Strongly disagree	Strongly disagree	Strongly disagree	Strongly disagree	Strongly disagree	Strongly disagree	Strongly disagree
	Agree	Agree	Agree	Agree	Agree	Agree	Agree
	Strongly agree	Strongly agree	Strongly agree	Strongly agree	Strongly agree	Strongly agree	Strongly agree
	Agree	Agree	Neutral	Neutral	Strongly agree	Strongly agree	Strongly agree
	Strongly disagree	Strongly disagree	Strongly disagree	Disagree	Strongly disagree	Strongly disagree	Strongly disagree
	Strongly agree	Strongly agree	Strongly agree	Strongly agree	Strongly agree	Strongly agree	Strongly agree
	Strongly agree	Agree	Disagree	Disagree	Strongly agree	Neutral	Strongly agree
	Strongly agree	Strongly agree	Strongly agree	Strongly agree	Strongly agree	Strongly agree	Strongly agree
	Strongly disagree	Strongly disagree	Strongly disagree	Strongly disagree	Strongly disagree	Strongly disagree	Strongly disagree
	Strongly disagree	Strongly disagree	Strongly disagree	Strongly disagree	Strongly disagree	Strongly disagree	Strongly disagree
	Agree	Agree	Agree	Agree	Agree	Agree	Agree

I advise pregnant women to recieve oral health information through WhatsApp	I will join WhatsApp groups that transmit useful oral health information.	Knowing that the oral health information were revied scentifically was important to me.	T3: Pregnant women cannot receive dental treatment at all, as it is unsafe.	T3: Pregnant women can receive emergency treatment such as root canal treatment or tooth extraction.	T3: The best time to receive emergency dental treatment is from the fourth month to the sixth month (second trimester)	T3: It is okay to receive non-emergency treatments, such as teeth whitening and cosmetic treatments in the last three months of pregnancy. (The last trimester)	T3: It is preferable that the appointments for visiting the dentist be short.	T3: Endo treatment is important to avoid complications such as inflammation and swelling that can affect the airway
			No	Yes	Yes	No	Yes	Yes
			No	Yes	Yes	No	Yes	Yes
			No	Yes	Yes	Yes	Yes	Yes
			No	Yes	Yes	No	Yes	Yes
			No	Yes	Yes	No	Yes	Yes
			No	Yes	No	No	I do not know	Yes
			No	Yes	Yes	Yes	No	Yes
			No	Yes	Yes	No	Yes	Yes
			No	Yes	Yes	No	Yes	Yes
			Yes	I do not know	I do not know	I do not know	Yes	Yes
			No	No	I do not know	No	No	I do not know
			No	Yes	Yes	No	Yes	Yes
			No	Yes	Yes	No	Yes	Yes
			Yes	I do not know	Yes	No	Yes	Yes
			No	Yes	Yes	No	Yes	Yes
			No	Yes	Yes	Yes	Yes	Yes
			No	Yes	Yes	No	Yes	Yes
			No	Yes	Yes	No	Yes	Yes
			No	Yes	Yes	Yes	No	Yes
			No	Yes	Yes	No	Yes	Yes
			No	Yes	Yes	No	Yes	Yes
			No	Yes	Yes	No	Yes	Yes
			No	Yes	Yes	No	Yes	Yes
			No	Yes	Yes	No	Yes	Yes
			No	Yes	Yes	No	Yes	Yes
			No	Yes	Yes	Yes	Yes	No
			No	Yes	Yes	No	Yes	Yes
			No	Yes	Yes	I do not know	I do not know	Yes
			No	No	Yes	Yes	Yes	Yes
			No	Yes	Yes	No	Yes	Yes
Strongly agree	Strongly agree	Strongly agree	No	Yes	Yes	I do not know	Yes	Yes
Strongly agree	Strongly agree	Strongly agree	No	Yes	Yes	No	Yes	Yes
Neutral	Strongly disagree	Strongly agree	No	Yes	Yes	No	No	Yes
Strongly agree	Agree	Strongly agree	No	Yes	Yes	No	Yes	Yes
Strongly disagree	Strongly disagree	Strongly disagree	No	Yes	Yes	No	Yes	Yes
Agree	Strongly disagree	Agree	No	Yes	Yes	No	Yes	Yes
Disagree	Disagree	Strongly agree	No	Yes	Yes	No	Yes	Yes
Strongly agree	Strongly agree	Strongly agree	No	Yes	Yes	No	Yes	I do not know
Agree	Neutral	Strongly agree	No	Yes	Yes	No	Yes	Yes
Agree	Agree	Agree	No	Yes	Yes	No	Yes	Yes
Strongly disagree	Strongly disagree	Strongly disagree	No	Yes	Yes	No	Yes	Yes
Agree	Agree	Agree	No	Yes	Yes	Yes	Yes	Yes
Strongly agree	Strongly agree	Strongly agree	No	Yes	Yes	Yes	Yes	Yes
Strongly disagree	Strongly disagree	Strongly disagree	No	Yes	Yes	No	Yes	Yes
Agree	Agree	Agree	No	Yes	Yes	Yes	Yes	Yes
Agree	Agree	Agree	No	Yes	Yes	No	No	Yes
Strongly agree	Strongly agree	Strongly agree	No	Yes	Yes	No	Yes	Yes
Disagree	Agree	Agree	No	Yes	Yes	Yes	Yes	I do not know
Strongly agree	Agree	Agree	No	Yes	Yes	Yes	No	Yes
Neutral	Disagree	Agree	No	Yes	Yes	No	Yes	Yes
Agree	Agree	Strongly agree	No	Yes	Yes	No	I do not know	Yes
Strongly disagree	Strongly disagree	Strongly disagree	No	Yes	Yes	Yes	Yes	Yes
Strongly disagree	Strongly disagree	Strongly disagree	No	Yes	Yes	No	Yes	Yes
Agree	Agree	Agree	No	No	No	Yes	I do not know	Yes
Strongly agree	Strongly agree	Strongly agree	No	No	Yes	No	Yes	Yes
Neutral	Neutral	Strongly agree	No	Yes	Yes	No	Yes	Yes
Strongly disagree	Strongly disagree	Disagree	No	Yes	Yes	No	Yes	Yes
Strongly agree	Strongly agree	Strongly agree	No	Yes	No	Yes	Yes	Yes
Disagree	Disagree	Strongly agree	No	Yes	Yes	No	Yes	Yes
Strongly agree	Strongly agree	Strongly agree	No	Yes	Yes	No	Yes	Yes
Strongly disagree	Strongly disagree	Strongly disagree	No	Yes	Yes	No	Yes	Yes
Strongly disagree	Strongly disagree	Strongly disagree	No	Yes	Yes	No	Yes	Yes
Neutral	Agree	Agree	No	Yes	Yes	No	Yes	Yes

T3: Most of the medicinal substances used to treat nerve pain are not safe for pregnant women	T3: The best position for a pregnant woman in the dental chair is a semi-reclined position with a pillow under the patient's right side.	T3: X-rays are not safe for pregnant women or infants even with necessary precautions.	T3: When a pregnant woman is exposed to X-rays, she should wear a lead urpon and a thyroid collar to protect the thyroid gland.	T3: X-rays are essential in various dental procedures, especially in emergency situations.	T3: Routine X-rays, usually taken during annual checkups, are best taken during pregnancy.	T3: Is it forbidden for pregnant women to use all types of dental local anesthetics	T3: The most common antibiotics, such as amoxicillin, clindamycin, metronidazole, and penicillin (High Musk, Amoxil), are safe for pregnant patients.	T3: Acetaminophen (paracetamol) such as Panadol and Vivadol: the safest analgesic for pregnant and breast-feeding patients.
No	Yes	No	Yes	Yes	Yes	No	Yes	Yes
No	Yes	No	Yes	Yes	Yes	No	Yes	Yes
No	Yes	No	Yes	Yes	No	No	Yes	Yes
No	Yes	No	Yes	No	No	No	Yes	Yes
No	Yes	No	Yes	Yes	I do not know	No	No	Yes
No	Yes	No	Yes	Yes	No	No	Yes	Yes
No	I do not know	Yes	I do not know	No	Yes	No	I do not know	Yes
No	Yes	Yes	Yes	Yes	No	No	No	Yes
No	Yes	No	Yes	Yes	No	I do not know	Yes	Yes
Yes	Yes	Yes	Yes	Yes	No	Yes	No	No
I do not know	Yes	No	Yes	Yes	No	Yes	Yes	Yes
No	Yes	No	Yes	No	Yes	No	No	Yes
No	Yes	No	Yes	Yes	I do not know	No	No	Yes
Yes	Yes	Yes	Yes	Yes	Yes	Yes	I do not know	I do not know
Yes	Yes	No	Yes	No	No	Yes	No	Yes
No	Yes	Yes	Yes	Yes	Yes	No	No	Yes
No	Yes	No	Yes	I do not know	No	No	Yes	Yes
No	Yes	No	Yes	Yes	No	No	No	Yes
No	Yes	No	Yes	Yes	Yes	No	Yes	Yes
Yes	Yes	Yes	Yes	Yes	Yes	Yes	Yes	Yes
No	Yes	No	Yes	Yes	No	No	No	Yes
Yes	Yes	Yes	Yes	Yes	Yes	Yes	I do not know	Yes
No	Yes	No	Yes	Yes	Yes	No	Yes	Yes
No	Yes	Yes	Yes	No	No	No	No	Yes
No	Yes	No	Yes	No	No	No	No	Yes
No	Yes	No	Yes	Yes	Yes	Yes	I do not know	Yes
No	Yes	No	Yes	No	No	No	No	Yes
I do not know	Yes	Yes	Yes	Yes	Yes	I do not know	I do not know	I do not know
No	No	Yes	I do not know	Yes	No	No	Yes	No
No	Yes	No	Yes	Yes	Yes	No	No	Yes
No	Yes	Yes	Yes	Yes	No	No	I do not know	Yes
No	No	Yes	Yes	No	No	No	Yes	No
I do not know	Yes	I do not know	Yes	Yes	No	I do not know	No	Yes
No	Yes	No	Yes	No	Yes	No	Yes	Yes
No	Yes	Yes	Yes	No	No	No	No	Yes
No	Yes	Yes	Yes	No	No	Yes	Yes	Yes
No	I do not know	No	I do not know	I do not know	I do not know	No	I do not know	Yes
Yes	Yes	Yes	I do not know	No	No	I do not know	Yes	Yes
No	Yes	No	No	Yes	Yes	No	Yes	Yes
No	Yes	No	Yes	Yes	No	No	Yes	Yes
No	Yes	No	Yes	No	Yes	No	Yes	Yes
No	Yes	No	Yes	Yes	I do not know	No	Yes	Yes
No	Yes	No	Yes	Yes	Yes	No	No	Yes
Yes	Yes	No	Yes	No	Yes	Yes	No	Yes
No	Yes	No	Yes	Yes	Yes	No	No	Yes
No	I do not know	Yes	I do not know	I do not know	I do not know	No	I do not know	I do not know
No	Yes	No	Yes	Yes	No	No	Yes	Yes
Yes	Yes	Yes	Yes	I do not know	I do not know	Yes	Yes	Yes
Yes	Yes	No	Yes	Yes	No	No	No	Yes
No	Yes	No	Yes	Yes	No	No	No	Yes
I do not know	Yes	Yes	Yes	I do not know	I do not know	No	I do not know	Yes
No	Yes	No	Yes	Yes	Yes	Yes	Yes	Yes
No	Yes	No	Yes	Yes	No	No	Yes	Yes
I do not know	Yes	I do not know	Yes	Yes	Yes	Yes	I do not know	Yes
Yes	Yes	Yes	Yes	Yes	Yes	No	Yes	Yes
No	Yes	No	Yes	Yes	No	No	Yes	Yes
No	Yes	No	Yes	Yes	I do not know	No	No	Yes
No	Yes	No	Yes	No	No	No	No	Yes
No	Yes	No	Yes	Yes	No	No	Yes	Yes
No	Yes	Yes	Yes	Yes	No	No	No	Yes
Yes	Yes	No	Yes	Yes	No	No	No	Yes
No	Yes	No	Yes	Yes	Yes	No	Yes	Yes
I do not know	Yes	I do not know	No	Yes	I do not know	No	No	Yes

T3: Non-steroidal anti-inflammatory drugs such as ibuprofen: safe during pregnancy (such as ibuprofen).	T3: Gingivitis usually increases during pregnancy from the fourth to the sixth month (second trimester); Which is known as: redness, swelling, bleeding, pain, difficulty chewing, movement of the teeth	T3: Preventive dental cleanings and annual exams during pregnancy are safe	T3: To reduce gum disease: brush your teeth twice with a soft brush and fluoride toothpaste and use dental floss	T3: Do not gargle with salt water	T1 marked: Pregnant women cannot receive dental treatment at all, as it is unsafe.	T1 marked: Pregnant women can receive emergency treatment such as root canal treatment or tooth extraction.	T1 marked: The best time to receive emergency dental treatment is from the fourth month to the sixth month (second trimester)	T1 marked: It is okay to receive non-emergency treatments, such as teeth whitening and cosmetic treatments in the last three months of pregnancy. (The last trimester)
No	Yes	Yes	Yes	Yes	Correct	Correct	Correct	Correct
No	Yes	Yes	Yes	No	Correct	Wrong	Correct	Correct
No	No	Yes	Yes	No	Correct	Correct	Correct	Wrong
No	Yes	Yes	Yes	No	Correct	Correct	Correct	Wrong
No	Yes	Yes	Yes	No	Correct	Wrong	Correct	Wrong
No	Yes	Yes	Yes	No	Wrong	Wrong	Wrong	Wrong
Yes	Yes	Yes	Yes	No	Wrong	Wrong	Correct	Wrong
No	Yes	Yes	Yes	No	Wrong	Correct	Correct	Correct
No	Yes	Yes	Yes	No	Wrong	Correct	Correct	Correct
No	Yes	I do not know	Yes	No	Wrong	Correct	Correct	Correct
Yes	No	Yes	Yes	No	Correct	Correct	Correct	Wrong
No	Yes	Yes	Yes	No	Correct	Correct	Wrong	Wrong
No	Yes	Yes	Yes	No	Correct	Wrong	Correct	Correct
I do not know	Yes	Yes	Yes	I do not know	Wrong	Wrong	Wrong	Correct
No	Yes	Yes	Yes	No	Wrong	Wrong	Wrong	Correct
No	Yes	Yes	Yes	No	Correct	Correct	Correct	Wrong
No	Yes	Yes	Yes	No	Correct	Correct	Correct	Wrong
No	Yes	Yes	Yes	No	Wrong	Wrong	Wrong	Correct
No	Yes	No	Yes	Yes	Correct	Correct	Correct	Correct
Yes	Yes	Yes	Yes	Yes	Correct	Correct	Correct	Correct
No	Yes	Yes	Yes	No	Correct	Correct	Correct	Wrong
No	Yes	Yes	Yes	No	Wrong	Correct	Wrong	Wrong
No	Yes	No	Yes	No	Correct	Wrong	Correct	Wrong
No	Yes	Yes	Yes	No	Correct	Correct	Correct	Correct
Yes	Yes	Yes	Yes	I do not know	Wrong	Wrong	Wrong	Wrong
I do not know	Yes	Yes	Yes	Yes	Correct	Correct	Wrong	Wrong
No	Yes	Yes	Yes	No	Correct	Wrong	Wrong	Wrong
I do not know	I do not know	Yes	Yes	No	Wrong	Wrong	Wrong	Correct
No	No	No	Yes	Yes	Correct	Correct	Correct	Correct
Yes	Yes	I do not know	Yes	No	Wrong	Wrong	Correct	Correct
No	Yes	Yes	Yes	No	Correct	Correct	Correct	Wrong
No	Yes	Yes	Yes	No	Correct	Correct	Correct	Correct
No	Yes	Yes	Yes	No	Correct	Wrong	Correct	Wrong
No	Yes	Yes	Yes	No	Correct	Correct	Wrong	Wrong
No	Yes	Yes	Yes	No	Wrong	Correct	Correct	Wrong
No	Yes	Yes	Yes	No	Wrong	Wrong	Wrong	Correct
I do not know	I do not know	Yes	Yes	No	Correct	Correct	Correct	Wrong
Yes	Yes	Yes	No	No	Correct	Correct	Wrong	Correct
No	Yes	Yes	Yes	No	Wrong	Wrong	Wrong	Wrong
No	Yes	Yes	Yes	No	Correct	Correct	Correct	Wrong
No	Yes	Yes	Yes	No	Correct	Correct	Correct	Wrong
No	Yes	Yes	Yes	No	Correct	Correct	Correct	Wrong
No	Yes	Yes	Yes	No	Correct	Correct	Correct	Correct
No	Yes	Yes	Yes	No	Wrong	Wrong	Wrong	Correct
No	Yes	Yes	Yes	No	Correct	Correct	Correct	Wrong
I do not know	I do not know	Yes	Yes	No	Correct	Correct	Correct	Correct
No	Yes	Yes	Yes	No	Correct	Correct	Correct	Wrong
Yes	Yes	I do not know	Yes	Yes	Wrong	Wrong	Correct	Wrong
No	Yes	Yes	Yes	No	Wrong	Correct	Correct	Wrong
No	Yes	Yes	Yes	No	Correct	Correct	Wrong	Correct
Yes	Yes	Yes	Yes	No	Correct	Wrong	Wrong	Correct
No	Yes	Yes	Yes	No	Correct	Correct	Wrong	Wrong
No	No	Yes	Yes	No	Correct	Correct	Wrong	Wrong
No	Yes	I do not know	Yes	I do not know	Correct	Wrong	Wrong	Wrong
No	Yes	Yes	Yes	No	Wrong	Wrong	Correct	Correct
I do not know	Yes	I do not know	Yes	No	Correct	Correct	Wrong	Wrong
Yes	Yes	I do not know	Yes	No	Wrong	Wrong	Wrong	Correct
No	Yes	Yes	Yes	No	Wrong	Wrong	Wrong	Wrong
No	Yes	Yes	Yes	No	Correct	Wrong	Wrong	Wrong
No	Yes	Yes	Yes	No	Correct	Correct	Correct	Correct
No	Yes	Yes	Yes	No	Correct	Correct	Wrong	Wrong
No	Yes	Yes	Yes	No	Correct	Correct	Correct	Wrong
No	Yes	Yes	Yes	No	Wrong	Wrong	Wrong	Wrong

T1 marked: It is preferable that the appointments for visiting the dentist be short.	T1 marked: Endo treatment is important to avoid complications such as inflammation and swelling that can affect the airway	T1 marked: Most of the medicinal substances used to treat nerve pain are not safe for pregnant women	T1 marked: The best position for a pregnant woman in the dental chair is a semi-reclined position with a pillow under the patient's right side.	T1 marked: X-rays are not safe for pregnant women or infants even with necessary precautions.	T1 marked: When a pregnant woman is exposed to X-rays, she should wear a lead urpon and a thyroid collar to protect the thyroid gland.	T1 marked: X-rays are essential in various dental procedures, especially in emergency situations.	T1 marked: Routine X-rays, usually taken during annual checkups, are best taken during pregnancy.	T1 marked: Is it forbidden for pregnant women to use all types of dental local anesthetics
Correct	Correct	Wrong	Wrong	Wrong	Wrong	Wrong	Correct	Correct
Correct	Correct	Wrong	Correct	Correct	Correct	Correct	Wrong	Correct
Wrong	Wrong	Wrong	Wrong	Wrong	Wrong	Wrong	Wrong	Correct
Correct	Correct	Wrong	Wrong	Correct	Correct	Correct	Correct	Correct
Wrong	Wrong	Wrong	Wrong	Wrong	Correct	Correct	Wrong	Wrong
Wrong	Wrong	Wrong	Wrong	Wrong	Correct	Correct	Wrong	Wrong
Wrong	Correct	Wrong	Wrong	Wrong	Wrong	Wrong	Wrong	Wrong
Wrong	Correct	Wrong	Wrong	Wrong	Correct	Correct	Correct	Wrong
Wrong	Wrong	Wrong	Correct	Wrong	Wrong	Correct	Correct	Wrong
Correct	Correct	Wrong	Wrong	Wrong	Correct	Correct	Correct	Wrong
Wrong	Correct	Wrong	Wrong	Wrong	Correct	Correct	Wrong	Correct
Wrong	Correct	Correct	Correct	Wrong	Correct	Wrong	Wrong	Correct
Wrong	Wrong	Wrong	Wrong	Wrong	Wrong	Wrong	Correct	Wrong
Correct	Correct	Wrong	Wrong	Wrong	Correct	Wrong	Correct	Wrong
Correct	Correct	Wrong	Correct	Wrong	Wrong	Wrong	Wrong	Wrong
Correct	Wrong	Correct	Correct	Correct	Correct	Correct	Correct	Wrong
Correct	Correct	Wrong	Correct	Wrong	Correct	Wrong	Correct	Correct
Correct	Correct	Wrong	Correct	Wrong	Wrong	Correct	Wrong	Wrong
Correct	Wrong	Wrong	Wrong	Wrong	Wrong	Wrong	Correct	Correct
Correct	Correct	Wrong	Correct	Wrong	Correct	Correct	Correct	Wrong
Wrong	Correct	Wrong	Wrong	Correct	Correct	Correct	Correct	Correct
Wrong	Correct	Wrong	Wrong	Wrong	Wrong	Wrong	Correct	Wrong
Wrong	Correct	Wrong	Wrong	Wrong	Correct	Wrong	Wrong	Wrong
Wrong	Wrong	Wrong	Wrong	Wrong	Wrong	Wrong	Correct	Wrong
Correct	Wrong	Wrong	Wrong	Wrong	Wrong	Correct	Wrong	Wrong
Wrong	Correct	Correct	Correct	Wrong	Correct	Correct	Correct	Correct
Wrong	Wrong	Wrong	Wrong	Wrong	Wrong	Wrong	Wrong	Wrong
Wrong	Wrong	Wrong	Wrong	Wrong	Correct	Correct	Wrong	Wrong
Wrong	Correct	Wrong	Wrong	Wrong	Correct	Correct	Wrong	Wrong
Correct	Wrong	Wrong	Correct	Wrong	Correct	Correct	Correct	Wrong
Correct	Correct	Wrong	Wrong	Wrong	Correct	Wrong	Wrong	Correct
Wrong	Correct	Correct	Wrong	Wrong	Correct	Correct	Wrong	Correct
Wrong	Correct	Wrong	Wrong	Wrong	Correct	Correct	Wrong	Wrong
Wrong	Correct	Correct	Wrong	Wrong	Wrong	Wrong	Wrong	Wrong
Correct	Correct	Wrong	Correct	Wrong	Correct	Wrong	Wrong	Correct
Correct	Correct	Wrong	Wrong	Wrong	Wrong	Wrong	Wrong	Wrong
Correct	Correct	Wrong	Correct	Wrong	Wrong	Wrong	Wrong	Wrong
Wrong	Wrong	Wrong	Wrong	Wrong	Wrong	Wrong	Correct	Wrong
Correct	Correct	Wrong	Wrong	Wrong	Wrong	Correct	Wrong	Wrong
Correct	Correct	Correct	Correct	Correct	Correct	Correct	Correct	Correct
Correct	Correct	Correct	Wrong	Correct	Wrong	Correct	Correct	Correct
Wrong	Correct	Correct	Correct	Correct	Correct	Wrong	Correct	Correct
Wrong	Wrong	Wrong	Wrong	Wrong	Wrong	Wrong	Wrong	Wrong
Wrong	Wrong	Wrong	Wrong	Wrong	Correct	Wrong	Correct	Correct
Correct	Wrong	Wrong	Correct	Wrong	Correct	Correct	Correct	Wrong
Correct	Correct	Correct	Wrong	Wrong	Wrong	Wrong	Wrong	Correct
Wrong	Wrong	Wrong	Wrong	Correct	Correct	Correct	Wrong	Correct
Correct	Wrong	Wrong	Wrong	Wrong	Wrong	Wrong	Wrong	Wrong
Correct	Correct	Wrong	Wrong	Wrong	Correct	Wrong	Wrong	Wrong
Correct	Correct	Wrong	Wrong	Wrong	Correct	Correct	Wrong	Correct
Correct	Correct	Correct	Correct	Wrong	Wrong	Wrong	Wrong	Correct
Wrong	Correct	Correct	Correct	Correct	Correct	Correct	Wrong	Correct
Correct	Correct	Correct	Wrong	Wrong	Correct	Wrong	Wrong	Correct
Wrong	Wrong	Wrong	Correct	Wrong	Wrong	Correct	Wrong	Wrong
Wrong	Correct	Wrong	Correct	Wrong	Correct	Correct	Wrong	Wrong
Wrong	Correct	Correct	Wrong	Wrong	Correct	Wrong	Correct	Correct
Correct	Correct	Wrong	Wrong	Wrong	Wrong	Wrong	Wrong	Wrong
Wrong	Correct	Wrong	Wrong	Wrong	Wrong	Wrong	Wrong	Wrong
Wrong	Correct	Wrong	Wrong	Wrong	Wrong	Wrong	Wrong	Wrong
Correct	Correct	Wrong	Wrong	Wrong	Wrong	Correct	Wrong	Correct
Wrong	Correct	Wrong	Wrong	Wrong	Wrong	Wrong	Wrong	Wrong
Wrong	Correct	Correct	Correct	Wrong	Wrong	Wrong	Wrong	Correct
Wrong	Wrong	Wrong	Wrong	Wrong	Wrong	Wrong	Wrong	Wrong

T1 marked: The most common antibiotics, such as amoxicillin, clindamycin, metronidazole, and penicillin (High Musk, Amoxil), are safe for pregnant patients.	T1 marked: Acetaminophen (paracetamol) such as Panadol and Vivadol: the safest analgesic for pregnant and breast-feeding patients.	T1 marked: Non-steroidal anti-inflammatory drugs such as ibuprofen: safe during pregnancy (such as ibuprofen).	T1 marked: Gingivitis usually increases during pregnancy from the fourth to the sixth month (second trimester); Which is known as: redness, swelling, bleeding, pain, difficulty chewing, movement of the teeth	T1 marked: Preventive dental cleanings and annual exams during pregnancy are safe	T1 marked: To reduce gum disease: brush your teeth twice with a soft brush and fluoride toothpaste and use dental floss	T1 marked: Do not gargle with salt water	T1 Total	T2: marked: Pregnant women cannot receive dental treatment at all, as it is unsafe.
Wrong	Correct	Correct	Wrong	Correct	Correct	Correct	13	Correct
Wrong	Correct	Wrong	Correct	Correct	Correct	Correct	15	Correct
Wrong	Correct	Wrong	Correct	Wrong	Wrong	Correct	7	Correct
Correct	Correct	Correct	Correct	Correct	Correct	Correct	17	Correct
Wrong	Wrong	Wrong	Correct	Correct	Correct	Correct	8	Correct
Wrong	Correct	Wrong	Wrong	Correct	Correct	Correct	6	Correct
Wrong	Wrong	Wrong	Correct	Correct	Correct	Correct	6	Correct
Wrong	Correct	Correct	Correct	Wrong	Correct	Correct	12	Correct
Wrong	Correct	Wrong	Correct	Correct	Correct	Correct	11	Correct
Wrong	Wrong	Correct	Correct	Correct	Correct	Correct	13	Wrong
Wrong	Correct	Correct	Correct	Correct	Correct	Correct	13	Correct
Wrong	Correct	Wrong	Wrong	Correct	Correct	Correct	11	Correct
Wrong	Correct	Correct	Wrong	Wrong	Correct	Correct	8	Correct
Wrong	Correct	Wrong	Correct	Wrong	Correct	Wrong	8	Wrong
Wrong	Correct	Wrong	Correct	Wrong	Wrong	Wrong	6	Correct
Correct	Wrong	Correct	Correct	Wrong	Wrong	Correct	14	Correct
Wrong	Correct	Wrong	Correct	Correct	Correct	Correct	14	Correct
Wrong	Correct	Wrong	Wrong	Wrong	Correct	Correct	8	Correct
Wrong	Correct	Wrong	Wrong	Correct	Correct	Correct	11	Correct
Wrong	Correct	Wrong	Correct	Correct	Correct	Correct	15	Wrong
Correct	Correct	Correct	Correct	Wrong	Correct	Correct	15	Correct
Wrong	Wrong	Correct	Wrong	Correct	Correct	Correct	7	Correct
Wrong	Correct	Wrong	Wrong	Correct	Correct	Correct	8	Correct
Wrong	Correct	Correct	Wrong	Correct	Correct	Correct	10	Correct
Wrong	Correct	Wrong	Wrong	Wrong	Correct	Wrong	4	Correct
Wrong	Correct	Wrong	Correct	Correct	Correct	Correct	14	Correct
Wrong	Correct	Wrong	Wrong	Wrong	Correct	Wrong	3	Correct
Wrong	Wrong	Correct	Correct	Correct	Correct	Correct	8	Correct
Wrong	Wrong	Wrong	Correct	Wrong	Wrong	Correct	9	Correct
Wrong	Correct	Correct	Wrong	Wrong	Correct	Correct	11	Correct
Wrong	Correct	Correct	Correct	Correct	Correct	Correct	13	Correct
Correct	Correct	Correct	Correct	Correct	Correct	Correct	16	Correct
Wrong	Correct	Correct	Correct	Correct	Correct	Wrong	10	Correct
Wrong	Correct	Correct	Correct	Correct	Correct	Correct	10	Correct
Wrong	Correct	Correct	Correct	Correct	Correct	Wrong	12	Correct
Correct	Correct	Correct	Correct	Correct	Correct	Correct	10	Correct
Wrong	Correct	Wrong	Wrong	Correct	Correct	Correct	10	Correct
Wrong	Correct	Correct	Correct	Correct	Correct	Correct	10	Correct
Wrong	Wrong	Wrong	Correct	Correct	Correct	Wrong	6	Correct
Wrong	Correct	Correct	Wrong	Correct	Correct	Correct	17	Correct
Correct	Correct	Wrong	Correct	Correct	Correct	Correct	16	Correct
Correct	Correct	Correct	Correct	Correct	Correct	Correct	17	Correct
Wrong	Wrong	Correct	Correct	Wrong	Correct	Correct	8	Correct
Wrong	Correct	Correct	Correct	Correct	Wrong	Correct	9	Correct
Wrong	Correct	Wrong	Correct	Correct	Correct	Correct	13	Correct
Wrong	Correct	Wrong	Wrong	Wrong	Wrong	Correct	10	Correct
Wrong	Correct	Correct	Wrong	Correct	Correct	Correct	12	Correct
Wrong	Correct	Wrong	Wrong	Correct	Correct	Wrong	5	Wrong
Wrong	Correct	Correct	Correct	Wrong	Correct	Correct	10	Correct
Wrong	Correct	Correct	Correct	Correct	Correct	Correct	14	Correct
Wrong	Correct	Wrong	Wrong	Correct	Correct	Correct	11	Correct
Wrong	Correct	Correct	Correct	Correct	Correct	Correct	15	Correct
Wrong	Correct	Wrong	Wrong	Correct	Correct	Correct	11	Correct
Wrong	Correct	Correct	Correct	Wrong	Wrong	Correct	7	Correct
Wrong	Correct	Correct	Correct	Correct	Correct	Correct	12	Correct
Correct	Correct	Wrong	Correct	Correct	Correct	Wrong	12	Correct
Wrong	Correct	Wrong	Wrong	Wrong	Correct	Correct	6	Correct
Wrong	Correct	Wrong	Correct	Wrong	Correct	Correct	5	Correct
Wrong	Correct	Wrong	Correct	Wrong	Correct	Correct	6	Correct
Wrong	Correct	Correct	Correct	Wrong	Correct	Correct	13	Correct
Wrong	Correct	Correct	Correct	Correct	Correct	Correct	9	Correct
Wrong	Correct	Correct	Correct	Correct	Correct	Correct	13	Correct
Wrong	Correct	Wrong	Wrong	Wrong	Correct	Correct	3	Correct

T2: marked: Pregnant women can receive emergency treatment such as root canal treatment or tooth extraction.	T2: marked: The best time to receive emergency dental treatment is from the fourth month to the sixth month (second trimester)	T2: marked: It is okay to receive non-emergency treatments, such as teeth whitening and cosmetic treatments in the last three months of pregnancy. (The last trimester)	T2: marked: It is preferable that the appointments for visiting the dentist be short.	T2: marked: Endo treatment is important to avoid complications such as inflammation and swelling that can affect the airway	T2: marked: Most of the medicinal substances used to treat nerve pain are not safe for pregnant women	T2: marked: The best position for a pregnant woman in the dental chair is a semi-reclined position with a pillow under the patient's right side.	T2: marked: X-rays are not safe for pregnant women or infants even with necessary precautions.	T2: marked: When a pregnant woman is exposed to X-rays, she should wear a lead urpon and a thyroid collar to protect the thyroid gland.
Correct	Correct	Correct	Correct	Correct	Wrong	Correct	Correct	Correct
Correct	Correct	Correct	Correct	Correct	Wrong	Correct	Correct	Correct
Correct	Correct	Correct	Correct	Correct	Correct	Wrong	Wrong	Correct
Correct	Correct	Correct	Correct	Correct	Correct	Correct	Correct	Correct
Correct	Correct	Correct	Correct	Correct	Correct	Correct	Correct	Correct
Correct	Wrong	Correct	Wrong	Correct	Correct	Correct	Correct	Correct
Correct	Correct	Wrong	Wrong	Correct	Correct	Correct	Wrong	Wrong
Correct	Correct	Wrong	Correct	Correct	Correct	Correct	Correct	Correct
Correct	Correct	Correct	Wrong	Correct	Correct	Correct	Wrong	Correct
Wrong	Wrong	Correct	Correct	Correct	Wrong	Wrong	Wrong	Correct
Wrong	Correct	Wrong	Correct	Wrong	Wrong	Correct	Wrong	Wrong
Correct	Correct	Correct	Correct	Correct	Correct	Correct	Correct	Correct
Correct	Correct	Correct	Correct	Correct	Correct	Correct	Correct	Correct
Correct	Wrong	Wrong	Correct	Correct	Wrong	Wrong	Wrong	Correct
Correct	Correct	Correct	Correct	Correct	Correct	Correct	Correct	Correct
Correct	Correct	Wrong	Correct	Wrong	Correct	Correct	Correct	Correct
Correct	Correct	Correct	Correct	Wrong	Correct	Correct	Correct	Correct
Correct	Correct	Correct	Correct	Correct	Correct	Correct	Correct	Correct
Correct	Correct	Wrong	Correct	Correct	Correct	Correct	Correct	Correct
Correct	Correct	Wrong	Wrong	Correct	Correct	Correct	Wrong	Correct
Correct	Correct	Correct	Wrong	Correct	Correct	Correct	Correct	Correct
Correct	Correct	Wrong	Correct	Correct	Wrong	Correct	Correct	Correct
Correct	Correct	Correct	Correct	Correct	Correct	Correct	Correct	Correct
Correct	Correct	Correct	Correct	Correct	Correct	Correct	Correct	Correct
Correct	Wrong	Correct	Correct	Correct	Correct	Correct	Correct	Correct
Correct	Correct	Correct	Correct	Correct	Wrong	Correct	Correct	Correct
Correct	Correct	Correct	Correct	Correct	Correct	Correct	Correct	Correct
Correct	Wrong	Wrong	Wrong	Wrong	Wrong	Wrong	Wrong	Correct
Correct	Correct	Wrong	Wrong	Correct	Wrong	Correct	Wrong	Correct
Correct	Correct	Correct	Correct	Correct	Correct	Correct	Correct	Correct
Correct	Correct	Wrong	Correct	Correct	Correct	Correct	Wrong	Correct
Correct	Correct	Correct	Correct	Correct	Correct	Wrong	Correct	Correct
Correct	Correct	Correct	Wrong	Correct	Wrong	Correct	Wrong	Correct
Correct	Correct	Correct	Correct	Correct	Correct	Correct	Correct	Correct
Correct	Correct	Correct	Correct	Correct	Wrong	Correct	Wrong	Correct
Correct	Correct	Correct	Correct	Correct	Correct	Correct	Correct	Correct
Correct	Correct	Correct	Correct	Correct	Correct	Correct	Correct	Wrong
Correct	Correct	Correct	Correct	Correct	Correct	Wrong	Correct	Correct
Correct	Correct	Correct	Correct	Correct	Correct	Correct	Correct	Wrong
Correct	Wrong	Wrong	Correct	Correct	Correct	Correct	Correct	Correct
Correct	Correct	Correct	Correct	Correct	Correct	Correct	Correct	Correct
Correct	Wrong	Wrong	Correct	Correct	Correct	Correct	Correct	Correct
Correct	Correct	Correct	Wrong	Correct	Correct	Correct	Correct	Correct
Correct	Correct	Correct	Correct	Correct	Correct	Wrong	Correct	Correct
Correct	Correct	Correct	Correct	Correct	Correct	Correct	Correct	Correct
Correct	Correct	Correct	Correct	Correct	Correct	Wrong	Correct	Correct
Correct	Correct	Correct	Correct	Correct	Correct	Correct	Correct	Correct
Correct	Correct	Correct	Correct	Wrong	Correct	Correct	Wrong	Correct
Correct	Correct	Correct	Wrong	Correct	Correct	Correct	Wrong	Correct
Correct	Correct	Correct	Correct	Correct	Correct	Correct	Correct	Correct
Correct	Correct	Correct	Wrong	Correct	Correct	Correct	Correct	Wrong
Correct	Correct	Wrong	Wrong	Correct	Correct	Correct	Correct	Correct
Correct	Correct	Correct	Correct	Correct	Correct	Correct	Correct	Correct
Wrong	Wrong	Wrong	Wrong	Wrong	Wrong	Correct	Wrong	Wrong
Correct	Correct	Correct	Correct	Wrong	Wrong	Correct	Wrong	Correct
Correct	Wrong	Correct	Correct	Correct	Correct	Correct	Correct	Correct
Correct	Correct	Correct	Correct	Correct	Correct	Correct	Correct	Correct
Correct	Wrong	Correct	Correct	Correct	Correct	Correct	Correct	Correct
Correct	Wrong	Correct	Correct	Correct	Correct	Correct	Correct	Correct
Correct	Correct	Correct	Correct	Correct	Correct	Correct	Wrong	Correct
Correct	Wrong	Correct	Correct	Correct	Correct	Wrong	Correct	Correct
Correct	Correct	Correct	Correct	Correct	Correct	Correct	Correct	Correct
Correct	Correct	Correct	Correct	Correct	Wrong	Correct	Correct	Correct

T2: marked: X-rays are essential in various dental procedures, especially in emergency situations.	T2: marked: Routine X-rays, usually taken during annual checkups, are best taken during pregnancy.	T2: marked: Is it forbidden for pregnant women to use all types of dental local anesthetics	T2: marked: The most common antibiotics, such as amoxicillin, clindamycin, metronidazole, and penicillin (High Musk, Amoxil), are safe for pregnant patients.	T2: marked: Acetaminophen (paracetamol) such as Panadol and Vivadol: the safest analgesic for pregnant and breast-feeding patients.	T2: marked: Non-steroidal anti-inflammatory drugs such as ibuprofen: safe during pregnancy (such as ibuprofen).	T2: marked: Gingivitis usually increases during pregnancy from the fourth to the sixth month (second trimester); Which is known as: redness, swelling, bleeding, pain, difficulty chewing, movement of the teeth	T2: marked: Preventive dental cleanings and annual exams during pregnancy are safe	T2: marked: To reduce gum disease: brush your teeth twice with a soft brush and fluoride toothpaste and use dental floss
Wrong	Correct	Correct	Wrong	Correct	Correct	Correct	Correct	Correct
Wrong	Wrong	Correct	Correct	Correct	Correct	Correct	Correct	Correct
Correct	Wrong	Correct	Correct	Correct	Correct	Correct	Wrong	Correct
Correct	Correct	Correct	Correct	Correct	Correct	Correct	Correct	Correct
Correct	Correct	Correct	Wrong	Correct	Correct	Correct	Correct	Correct
Correct	Correct	Correct	Correct	Correct	Correct	Correct	Correct	Correct
Wrong	Correct	Correct	Wrong	Correct	Correct	Correct	Correct	Correct
Correct	Wrong	Correct	Correct	Correct	Correct	Correct	Correct	Correct
Correct	Correct	Correct	Wrong	Correct	Wrong	Correct	Correct	Correct
Correct	Wrong	Wrong	Wrong	Wrong	Correct	Correct	Correct	Correct
Wrong	Wrong	Wrong	Wrong	Correct	Correct	Wrong	Correct	Correct
Wrong	Correct	Correct	Wrong	Correct	Correct	Correct	Correct	Correct
Correct	Wrong	Correct	Wrong	Correct	Correct	Correct	Correct	Correct
Wrong	Wrong	Correct	Wrong	Correct	Wrong	Correct	Wrong	Correct
Correct	Correct	Correct	Wrong	Correct	Correct	Correct	Correct	Correct
Correct	Correct	Correct	Wrong	Correct	Correct	Correct	Correct	Correct
Wrong	Wrong	Correct	Correct	Correct	Correct	Correct	Correct	Correct
Correct	Correct	Correct	Correct	Correct	Correct	Correct	Correct	Correct
Correct	Correct	Correct	Wrong	Correct	Correct	Correct	Wrong	Correct
Correct	Wrong	Wrong	Wrong	Correct	Wrong	Correct	Correct	Correct
Correct	Correct	Correct	Correct	Correct	Correct	Correct	Correct	Correct
Correct	Wrong	Correct	Wrong	Correct	Correct	Correct	Correct	Correct
Wrong	Wrong	Correct	Correct	Correct	Correct	Correct	Correct	Correct
Wrong	Correct	Correct	Wrong	Correct	Wrong	Correct	Correct	Correct
Wrong	Wrong	Correct	Correct	Correct	Correct	Correct	Correct	Correct
Correct	Correct	Correct	Wrong	Correct	Correct	Correct	Correct	Correct
Wrong	Correct	Correct	Correct	Correct	Correct	Correct	Correct	Correct
Correct	Wrong	Wrong	Wrong	Wrong	Wrong	Wrong	Correct	Correct
Wrong	Wrong	Wrong	Wrong	Correct	Correct	Correct	Correct	Correct
Correct	Wrong	Correct	Wrong	Correct	Correct	Correct	Correct	Correct
Wrong	Wrong	Correct	Correct	Correct	Correct	Correct	Correct	Correct
Correct	Correct	Correct	Correct	Correct	Wrong	Correct	Correct	Correct
Correct	Correct	Correct	Wrong	Correct	Correct	Correct	Correct	Correct
Wrong	Correct	Correct	Correct	Correct	Correct	Correct	Correct	Correct
Wrong	Correct	Correct	Wrong	Correct	Correct	Correct	Correct	Correct
Wrong	Correct	Correct	Correct	Correct	Wrong	Correct	Correct	Correct
Wrong	Wrong	Correct	Wrong	Correct	Correct	Wrong	Wrong	Correct
Correct	Correct	Correct	Correct	Correct	Correct	Correct	Correct	Correct
Wrong	Correct	Correct	Correct	Wrong	Correct	Correct	Correct	Correct
Correct	Correct	Correct	Correct	Correct	Correct	Correct	Correct	Correct
Wrong	Wrong	Correct	Correct	Correct	Correct	Correct	Correct	Correct
Wrong	Correct	Correct	Correct	Correct	Correct	Correct	Correct	Correct
Wrong	Correct	Wrong	Correct	Correct	Correct	Correct	Correct	Correct
Correct	Correct	Correct	Wrong	Correct	Correct	Correct	Correct	Correct
Wrong	Correct	Correct	Correct	Correct	Wrong	Correct	Correct	Correct
Correct	Correct	Wrong	Correct	Correct	Wrong	Correct	Correct	Correct
Correct	Correct	Correct	Correct	Correct	Correct	Correct	Correct	Correct
Wrong	Wrong	Wrong	Correct	Correct	Correct	Correct	Correct	Correct
Correct	Wrong	Correct	Correct	Wrong	Correct	Correct	Correct	Correct
Correct	Correct	Correct	Correct	Correct	Correct	Correct	Correct	Correct
Wrong	Correct	Correct	Wrong	Correct	Wrong	Correct	Correct	Correct
Correct	Wrong	Correct	Correct	Correct	Correct	Correct	Correct	Correct
Correct	Correct	Correct	Correct	Correct	Correct	Wrong	Correct	Correct
Wrong	Wrong	Wrong	Wrong	Correct	Wrong	Correct	Correct	Correct
Wrong	Correct	Correct	Wrong	Correct	Correct	Correct	Wrong	Correct
Wrong	Correct	Correct	Correct	Correct	Correct	Correct	Wrong	Correct
Correct	Correct	Correct	Correct	Correct	Wrong	Correct	Correct	Correct
Wrong	Correct	Correct	Correct	Correct	Wrong	Correct	Correct	Correct
Correct	Correct	Correct	Correct	Correct	Correct	Correct	Correct	Correct
Correct	Wrong	Correct	Wrong	Correct	Correct	Correct	Correct	Correct
Wrong	Correct	Correct	Correct	Correct	Correct	Correct	Correct	Correct
Correct	Wrong	Correct	Correct	Correct	Correct	Correct	Correct	Correct
Correct	Correct	Wrong	Wrong	Correct	Correct	Correct	Wrong	Correct

T2: marked: Do not gargle with salt water	T2 Total	T3 marked: Pregnant women cannot receive dental treatment at all, as it is unsafe.	T3 marked: Pregnant women can receive emergency treatment such as root canal treatment or tooth extraction.	T3 marked: The best time to receive emergency dental treatment is from the fourth month to the sixth month (second trimester)	T3 marked: It is okay to receive non-emergency treatments, such as teeth whitening and cosmetic treatments in the last three months of pregnancy. (The last trimester)	T3 marked: It is preferable that the appointments for visiting the dentist be short.	T3 marked: Endo treatment is important to avoid complications such as inflammation and swelling that can affect the airway	T3 marked: Most of the medicinal substances used to treat nerve pain are not safe for pregnant women
Correct	17	Correct	Correct	Correct	Correct	Correct	Correct	Correct
Correct	17	Correct	Correct	Correct	Correct	Correct	Correct	Correct
Correct	16	Correct	Correct	Correct	Wrong	Correct	Correct	Correct
Correct	20	Correct	Correct	Correct	Correct	Correct	Correct	Correct
Correct	19	Correct	Correct	Correct	Correct	Correct	Correct	Correct
Correct	18	Correct	Correct	Wrong	Correct	Wrong	Correct	Correct
Correct	14	Correct	Correct	Correct	Wrong	Wrong	Correct	Correct
Correct	18	Correct	Correct	Correct	Correct	Correct	Correct	Correct
Correct	16	Correct	Correct	Correct	Correct	Correct	Correct	Correct
Correct	10	Wrong	Wrong	Wrong	Wrong	Correct	Correct	Wrong
Correct	9	Correct	Wrong	Wrong	Correct	Wrong	Wrong	Wrong
Correct	18	Correct	Correct	Correct	Correct	Correct	Correct	Correct
Correct	18	Correct	Correct	Correct	Correct	Correct	Correct	Correct
Correct	9	Wrong	Wrong	Correct	Correct	Correct	Correct	Wrong
Correct	19	Correct	Correct	Correct	Correct	Correct	Correct	Wrong
Correct	17	Correct	Correct	Correct	Wrong	Correct	Correct	Correct
Correct	17	Correct	Correct	Correct	Correct	Correct	Correct	Correct
Correct	20	Correct	Correct	Correct	Correct	Correct	Correct	Correct
Wrong	16	Correct	Correct	Correct	Wrong	Wrong	Correct	Correct
Correct	12	Correct	Correct	Correct	Correct	Correct	Correct	Wrong
Correct	19	Correct	Correct	Correct	Correct	Correct	Correct	Correct
Correct	16	Correct	Correct	Correct	Correct	Correct	Correct	Wrong
Correct	18	Correct	Correct	Correct	Correct	Correct	Correct	Correct
Correct	17	Correct	Correct	Correct	Correct	Correct	Correct	Correct
Correct	17	Correct	Correct	Correct	Correct	Correct	Correct	Correct
Correct	18	Correct	Correct	Correct	Wrong	Correct	Wrong	Correct
Correct	19	Correct	Correct	Correct	Correct	Correct	Correct	Correct
Correct	7	Correct	Correct	Correct	Wrong	Wrong	Correct	Wrong
Correct	12	Correct	Wrong	Correct	Wrong	Correct	Correct	Correct
Correct	18	Correct	Correct	Correct	Correct	Correct	Correct	Correct
Correct	16	Correct	Correct	Correct	Wrong	Correct	Correct	Correct
Correct	18	Correct	Correct	Correct	Correct	Correct	Correct	Correct
Correct	16	Correct	Correct	Correct	Correct	Wrong	Correct	Wrong
Correct	19	Correct	Correct	Correct	Correct	Correct	Correct	Correct
Correct	16	Correct	Correct	Correct	Correct	Correct	Correct	Correct
Correct	18	Correct	Correct	Correct	Correct	Correct	Correct	Correct
Correct	14	Correct	Correct	Correct	Correct	Correct	Correct	Correct
Correct	19	Correct	Correct	Correct	Correct	Correct	Wrong	Wrong
Correct	17	Correct	Correct	Correct	Correct	Correct	Correct	Correct
Correct	18	Correct	Correct	Correct	Correct	Correct	Correct	Correct
Correct	18	Correct	Correct	Correct	Correct	Correct	Correct	Correct
Correct	17	Correct	Correct	Correct	Wrong	Correct	Correct	Correct
Correct	17	Correct	Correct	Correct	Wrong	Correct	Correct	Correct
Correct	18	Correct	Correct	Correct	Correct	Correct	Correct	Wrong
Correct	18	Correct	Correct	Correct	Wrong	Correct	Correct	Correct
Correct	17	Correct	Correct	Correct	Correct	Wrong	Correct	Correct
Correct	20	Correct	Correct	Correct	Correct	Correct	Correct	Correct
Correct	14	Correct	Correct	Correct	Wrong	Correct	Wrong	Wrong
Correct	16	Correct	Correct	Correct	Wrong	Wrong	Correct	Wrong
Correct	20	Correct	Correct	Correct	Correct	Correct	Correct	Correct
Correct	15	Correct	Correct	Correct	Correct	Wrong	Correct	Wrong
Correct	17	Correct	Correct	Correct	Wrong	Correct	Correct	Correct
Correct	19	Correct	Correct	Correct	Correct	Correct	Correct	Correct
Wrong	6	Correct	Wrong	Wrong	Wrong	Wrong	Correct	Wrong
Correct	14	Correct	Wrong	Correct	Correct	Correct	Correct	Wrong
Correct	17	Correct	Correct	Correct	Correct	Correct	Correct	Correct
Correct	19	Correct	Correct	Correct	Correct	Correct	Correct	Correct
Correct	17	Correct	Correct	Wrong	Wrong	Correct	Correct	Correct
Correct	19	Correct	Correct	Correct	Correct	Correct	Correct	Correct
Correct	17	Correct	Correct	Correct	Correct	Correct	Correct	Correct
Correct	17	Correct	Correct	Correct	Correct	Correct	Correct	Wrong
Correct	19	Correct	Correct	Correct	Correct	Correct	Correct	Correct
Correct	16	Correct	Correct	Correct	Correct	Correct	Correct	Wrong

T3 marked: The best position for a pregnant woman in the dental chair is a semi-reclined position with a pillow under the patient's right side.	T3 marked: X-rays are not safe for pregnant women or infants even with necessary precautions.	T3 marked: When a pregnant woman is exposed to X-rays, she should wear a lead urpon and a thyroid collar to protect the thyroid gland.	T3 marked: X-rays are essential in various dental procedures, especially in emergency situations.	T3 marked: Routine X-rays, usually taken during annual checkups, are best taken during pregnancy.	T3 marked: Is it forbidden for pregnant women to use all types of dental local anesthetics	T3 marked: The most common antibiotics, such as amoxicillin, clindamycin, metronidazole, and penicillin (High Musk, Amoxil), are safe for pregnant patients.	T3 marked: Acetaminophen (paracetamol) such as Panadol and Vivadol: the safest analgesic for pregnant and breast-feeding patients.	T3 marked: Non-steroidal anti-inflammatory drugs such as ibuprofen: safe during pregnancy (such as ibuprofen).
Correct	Correct	Correct	Correct	Wrong	Correct	Correct	Correct	Correct
Correct	Correct	Correct	Correct	Wrong	Correct	Correct	Correct	Correct
Correct	Correct	Correct	Correct	Correct	Correct	Correct	Correct	Correct
Correct	Correct	Correct	Wrong	Correct	Correct	Correct	Correct	Correct
Correct	Correct	Correct	Correct	Wrong	Correct	Wrong	Correct	Correct
Correct	Correct	Correct	Correct	Correct	Correct	Correct	Correct	Correct
Wrong	Wrong	Wrong	Wrong	Wrong	Correct	Wrong	Correct	Wrong
Correct	Wrong	Correct	Correct	Correct	Correct	Wrong	Correct	Correct
Correct	Correct	Correct	Correct	Correct	Wrong	Correct	Correct	Correct
Correct	Wrong	Correct	Correct	Correct	Wrong	Wrong	Wrong	Correct
Correct	Correct	Correct	Correct	Correct	Wrong	Correct	Correct	Wrong
Correct	Correct	Correct	Wrong	Wrong	Correct	Wrong	Correct	Correct
Correct	Correct	Correct	Correct	Wrong	Correct	Wrong	Correct	Correct
Correct	Wrong	Correct	Correct	Wrong	Wrong	Wrong	Wrong	Wrong
Correct	Correct	Correct	Wrong	Correct	Wrong	Wrong	Correct	Correct
Correct	Wrong	Correct	Correct	Wrong	Correct	Wrong	Correct	Correct
Correct	Correct	Correct	Wrong	Correct	Correct	Correct	Correct	Correct
Correct	Correct	Correct	Correct	Correct	Correct	Wrong	Correct	Correct
Correct	Correct	Correct	Correct	Wrong	Correct	Correct	Correct	Correct
Correct	Wrong	Correct	Correct	Wrong	Wrong	Correct	Correct	Wrong
Correct	Correct	Correct	Correct	Correct	Correct	Wrong	Correct	Correct
Correct	Wrong	Correct	Correct	Wrong	Wrong	Wrong	Correct	Correct
Correct	Correct	Correct	Correct	Wrong	Correct	Correct	Correct	Correct
Correct	Wrong	Correct	Wrong	Correct	Correct	Wrong	Correct	Correct
Correct	Correct	Correct	Wrong	Correct	Correct	Wrong	Correct	Wrong
Correct	Correct	Correct	Correct	Wrong	Wrong	Wrong	Correct	Wrong
Correct	Correct	Correct	Wrong	Correct	Correct	Wrong	Correct	Correct
Correct	Wrong	Correct	Correct	Wrong	Wrong	Wrong	Wrong	Wrong
Wrong	Wrong	Wrong	Correct	Correct	Correct	Correct	Wrong	Correct
Correct	Correct	Correct	Correct	Wrong	Correct	Wrong	Correct	Wrong
Correct	Wrong	Correct	Correct	Correct	Correct	Wrong	Correct	Correct
Wrong	Wrong	Correct	Wrong	Correct	Correct	Correct	Wrong	Correct
Correct	Wrong	Correct	Correct	Correct	Wrong	Wrong	Correct	Correct
Correct	Correct	Correct	Wrong	Wrong	Correct	Correct	Correct	Correct
Correct	Wrong	Correct	Wrong	Correct	Correct	Wrong	Correct	Correct
Correct	Wrong	Correct	Wrong	Correct	Wrong	Correct	Correct	Correct
Wrong	Correct	Wrong	Wrong	Wrong	Correct	Wrong	Correct	Wrong
Correct	Wrong	Wrong	Wrong	Correct	Wrong	Correct	Correct	Wrong
Correct	Correct	Wrong	Correct	Wrong	Correct	Correct	Correct	Correct
Correct	Correct	Correct	Correct	Correct	Correct	Correct	Correct	Correct
Correct	Correct	Correct	Wrong	Wrong	Correct	Correct	Correct	Correct
Correct	Correct	Correct	Correct	Wrong	Correct	Correct	Correct	Correct
Correct	Correct	Correct	Correct	Wrong	Correct	Wrong	Correct	Correct
Correct	Correct	Correct	Wrong	Wrong	Wrong	Wrong	Correct	Correct
Correct	Correct	Correct	Correct	Wrong	Correct	Wrong	Correct	Correct
Wrong	Wrong	Wrong	Wrong	Wrong	Correct	Wrong	Wrong	Wrong
Correct	Correct	Correct	Correct	Correct	Correct	Correct	Correct	Correct
Correct	Wrong	Correct	Wrong	Wrong	Wrong	Correct	Correct	Wrong
Correct	Correct	Correct	Correct	Correct	Correct	Wrong	Correct	Correct
Correct	Correct	Correct	Correct	Correct	Correct	Wrong	Correct	Correct
Correct	Wrong	Correct	Wrong	Wrong	Correct	Wrong	Correct	Wrong
Correct	Correct	Correct	Correct	Wrong	Wrong	Correct	Correct	Correct
Correct	Correct	Correct	Correct	Correct	Correct	Correct	Correct	Correct
Correct	Wrong	Correct	Correct	Wrong	Wrong	Wrong	Correct	Correct
Correct	Wrong	Correct	Correct	Wrong	Correct	Correct	Correct	Correct
Correct	Correct	Correct	Correct	Correct	Correct	Correct	Correct	Wrong
Correct	Correct	Correct	Correct	Wrong	Correct	Wrong	Correct	Wrong
Correct	Correct	Correct	Wrong	Correct	Correct	Wrong	Correct	Correct
Correct	Correct	Correct	Correct	Correct	Correct	Correct	Correct	Correct
Correct	Wrong	Correct	Correct	Correct	Correct	Wrong	Correct	Correct
Correct	Correct	Correct	Correct	Correct	Correct	Wrong	Correct	Correct
Correct	Correct	Correct	Correct	Wrong	Correct	Correct	Correct	Correct
Correct	Wrong	Wrong	Correct	Wrong	Correct	Wrong	Correct	Correct

T3 marked: Gingivitis usually increases during pregnancy from the fourth to the sixth month (second trimester); Which is known as: redness, swelling, bleeding, pain, difficulty chewing, movement of the teeth	T3 marked: Preventive dental cleanings and annual exams during pregnancy are safe	T3 marked: To reduce gum disease: brush your teeth twice with a soft brush and fluoride toothpaste and use dental floss	T3 marked: Do not gargle with salt water	T3 Total	T2_T1	T3_T2	T3_T1
Correct	Correct	Correct	Wrong	18	4	1	5
Correct	Correct	Correct	Correct	19	2	2	4
Wrong	Correct	Correct	Correct	18	9	2	11
Correct	Correct	Correct	Correct	19	3	-1	2
Correct	Correct	Correct	Correct	18	11	-1	10
Correct	Correct	Correct	Correct	18	12	0	12
Correct	Correct	Correct	Correct	11	8	-3	5
Correct	Correct	Correct	Correct	18	6	0	6
Correct	Correct	Correct	Correct	19	5	3	8
Correct	Wrong	Correct	Correct	10	-3	0	-3
Wrong	Correct	Correct	Correct	12	-4	3	-1
Correct	Correct	Correct	Correct	17	7	-1	6
Correct	Correct	Correct	Correct	18	10	0	10
Correct	Correct	Correct	Wrong	10	1	1	2
Correct	Correct	Correct	Correct	16	13	-3	10
Correct	Correct	Correct	Correct	16	3	-1	2
Correct	Correct	Correct	Correct	19	3	2	5
Correct	Correct	Correct	Correct	19	12	-1	11
Correct	Wrong	Correct	Wrong	15	5	-1	4
Correct	Correct	Correct	Wrong	14	-3	2	-1
Correct	Correct	Correct	Correct	19	4	0	4
Correct	Correct	Correct	Correct	15	9	-1	8
Correct	Wrong	Correct	Correct	18	10	0	10
Correct	Correct	Correct	Correct	17	7	0	7
Correct	Correct	Correct	Wrong	16	13	-1	12
Correct	Correct	Correct	Wrong	13	4	-5	-1
Correct	Correct	Correct	Correct	18	16	-1	15
Wrong	Correct	Correct	Correct	10	-1	3	2
Wrong	Wrong	Correct	Wrong	11	3	-1	2
Correct	Wrong	Correct	Correct	16	7	-2	5
Correct	Correct	Correct	Correct	17	3	1	4
Correct	Correct	Correct	Correct	16	2	-2	0
Correct	Correct	Correct	Correct	15	6	-1	5
Correct	Correct	Correct	Correct	18	9	-1	8
Correct	Correct	Correct	Correct	17	4	1	5
Correct	Correct	Correct	Correct	17	8	-1	7
Wrong	Correct	Correct	Correct	13	4	-1	3
Correct	Correct	Wrong	Correct	12	9	-7	2
Correct	Correct	Correct	Correct	18	11	1	12
Correct	Correct	Correct	Correct	20	1	2	3
Correct	Correct	Correct	Correct	18	2	0	2
Correct	Correct	Correct	Correct	18	0	1	1
Correct	Correct	Correct	Correct	17	9	0	9
Correct	Correct	Correct	Correct	15	9	-3	6
Correct	Correct	Correct	Correct	17	5	-1	4
Wrong	Correct	Correct	Correct	10	7	-7	0
Correct	Correct	Correct	Correct	20	8	0	8
Correct	Wrong	Correct	Wrong	10	9	-4	5
Correct	Correct	Correct	Correct	16	6	0	6
Correct	Correct	Correct	Correct	19	6	-1	5
Correct	Correct	Correct	Correct	13	4	-2	2
Correct	Correct	Correct	Correct	17	2	0	2
Wrong	Correct	Correct	Correct	19	8	0	8
Correct	Wrong	Correct	Wrong	9	-1	3	2
Correct	Correct	Correct	Correct	16	2	2	4
Correct	Wrong	Correct	Correct	18	5	1	6
Correct	Wrong	Correct	Correct	16	13	-3	10
Correct	Correct	Correct	Correct	16	12	-1	11
Correct	Correct	Correct	Correct	20	13	1	14
Correct	Correct	Correct	Correct	18	4	1	5
Correct	Correct	Correct	Correct	18	8	1	9
Correct	Correct	Correct	Correct	19	6	0	6
Correct	Correct	Correct	Correct	15	13	-1	12
